# Proteomic profiling reveals immunologic mediators of endotoxin-related lung function decline: a longitudinal prospective study in textile workers

**DOI:** 10.1186/s12931-026-03669-4

**Published:** 2026-04-17

**Authors:** Hantao Wang, Gabriel Goodney, Fengying Zhang, Peggy S. Lai, Li Su, Peng Gao, Jungeun Lim, Shelton Lo, Zhonghua Liu, David C. Christiani, Jason Y. Y. Wong

**Affiliations:** 1 Epidemiology and Community Health Branch, Division of Intramural Research, National Heart, Lung, and Blood Institute, National Institutes of Health, 10 Center Drive, Bethesda, MD 20892 USA; 2 Shanghai Putuo District People’s Hospital, 1291 Jiangning Rd, Putuo District, Shanghai, China; 3 Harvard Medical School, 25 Shattuck St, Boston, MA 02115 USA; 4 Massachusetts General Hospital, 55 Fruit Street, Boston, MA 02114 USA; 5 Department of Environmental Health, Harvard T.H. Chan School of Public Health, 665 Huntington Avenue, Boston, MA 02115 USA; 6 Department of Biostatistics, Mailman School of Public Health, Columbia University, 722 W 168th St, New York, NY 10032 USA

**Keywords:** Cotton dust, Occupational health, Occupational epidemiology, Vegetable dust

## Abstract

**Background:**

Endotoxin is a major pathogenic component of cotton dust in the textile industry. While airborne endotoxin is associated with lung function decline, the underlying molecular mechanisms remain unclear. Proteomic profiling could provide important insights into the pathways that drive the harmful respiratory effects of endotoxin.

**Methods:**

We conducted serum proteomic profiling of 221 endotoxin-exposed cotton workers and 192 endotoxin-free silk workers from the longitudinal cohort of the Shanghai Textile Worker Study. Using blood samples collected in 2016, proteins were quantified by data-independent acquisition mass spectrometry and genotyping was assessed using low-pass whole genome sequencing. Forced expiratory volume in 1 s (FEV-1) was measured in 2011 and 2016. We used adjusted regression to identify differentially expressed proteins (DEPs) between the exposed and control groups. Causal mediation analyses were performed to identify protein mediators. Mendelian Randomization (MR) analyses provided causal estimates of protein effects on lung function change.

**Results:**

Among 2,962 quantified proteins, we identified 224 proteins that were differentially expressed between cotton and silk workers (Bonferroni *p* < 0.05). Top enriched pathways associated with DEPs were the complement and coagulation cascades (KEGG: hsa04610) and the chemokine signaling pathway (KEGG: hsa04062). Adaptive immune proteins collectively mediated endotoxin-related lung function decline (binomial *p* = 9.63 × 10⁻⁹⁷). Two immunoglobulin domain proteins significantly (Bonferroni *p* < 0.05) mediated endotoxin effects on lung function change: Epididymis luminal protein 180 (HEL180) and IGL c3728_light_IGKV4-1_IGKJ1 (IGL c3728), with 46.4% (*p* = 0.04) and 46.9% (*p* = 0.05) proportion of the total effect that was mediated. MR estimates demonstrated that every 2-fold decrease in HEL180 and IGL c3728 expression was associated with an FEV-1 decline of 1.90 ml/year (95% CI: [0.79, 3.02]) and 2.36 ml/year (95% CI: [1.68, 3.02]), respectively.

**Conclusion:**

Using a trans-omic approach, our findings suggest that chronic endotoxin exposure suppresses immunoglobulin domain proteins, weakens adaptive immunity, and accelerates lung function decline. These findings provide greater precision in understanding biological mechanisms underlying endotoxin-related respiratory dysfunction.

**Supplementary Information:**

The online version contains supplementary material available at 10.1186/s12931-026-03669-4.

## Background

Textile manufacturing is one of the largest traditional industries worldwide employing approximately 430 million people [1]. Since the 1970 s, textile manufacturing has increasingly shifted to low- and middle-income countries (LMICs) as the rise of “fast fashion”, characterized by rapid production cycles and the frequent new collections, has intensified production demands [2]. Given that cotton is the most commonly used fiber in the textile industry, identifying and mitigating occupational health and safety risks is critically important, particularly in LMICs with less stringent labor regulations. As reported by the Economic Research Service of the U.S. Department of Agriculture, about 25% of all textile products are made of cotton. Cotton textile production involves organic dust, particularly during yarn preparation and processing. Textile workers encountering organic dust with elevated levels of bacterial endotoxin are at greater risk of respiratory conditions.

The primary toxicant in cotton dust is endotoxin or lipopolysaccharide (LPS), which is often found in the outer membrane of gram-negative bacteria. Endotoxin signals through Toll-like receptor 4 (TLR4) and triggers signaling pathways that involve the adaptor protein MyD88. This leads to NF-κB activation and the production of pro-inflammatory cytokines [3–5]. Our previous studies on textile workers in Shanghai, China [6–11] have linked endotoxin exposure to lung function decline. The association between endotoxin and adverse health effects is consistent with findings from other occupational studies of textile workers conducted worldwide [12], especially in LMICs [13]. These studies suggest that endotoxin triggers airway inflammation and hyper-responsiveness, ultimately leading to airway obstruction. Occupational exposure to endotoxin is not unique to textile workers. Individuals in other workplaces with similar organic dust exposures, such as animal feeding operations [14], are also highly exposed to endotoxin. Although our previous studies have examined how protein expression affects lung function [15], the molecular mechanisms by which proteomic features mediate the effects of chronic endotoxin exposure on lung function decline remain unclear.

In this study, we first identified biological pathways involved in chronic endotoxin exposure by comparing protein expression between endotoxin-exposed cotton workers and endotoxin-free silk workers. Next, we investigated how specific protein features causally mediated the effect of endotoxin on changes in lung function. Finally, by integrating genomic and proteomics data, we estimated how changes in protein mediator expression levels affected lung function.

## Methods

### Study design and study population

The Shanghai Textile Worker Study is a longitudinal prospective cohort study initiated in 1981, consisting of 447 cotton workers and 472 silk workers residing in Shanghai, China. Questionnaires on work history, respiratory symptoms, smoking history, and demographics were collected every five years along with spirometry tests. Details of the cohort are outlined in our supplementary materials and have been reported previously [6–11]. Ethical approval was obtained from the Institutional Review Boards of the Harvard School of Public Health and Shanghai Putuo District People’s Hospital.

### Exposure assessment of endotoxin

Area full-shift samples of cotton dust were collected on-site during each survey until the mill closures in 2001. Endotoxin concentrations were analyzed using the Limulus Amebocyte Lysate assay [6–11]. The cumulative endotoxin concentration (EU/m³) was approximated using job titles and work history.

### Respiratory outcome assessment

Forced expiratory volume in 1 s (FEV-1) was measured according to the spirometry technical standards of the American Thoracic Society (ATS) [16]. The primary outcome was the annual change in FEV-1 between the 2011 and 2016 surveys (ml/year), with positive values indicating an improvement in lung function and negative values indicating a decline.

### Proteomics

Serum samples were collected from 221 endotoxin-exposed cotton workers and 216 endotoxin-free silk workers in 2016. Protein quantification was performed using data-independent acquisition mass spectrometry (DIA-MS). Details of sample preparation can be found in our previous study [15] and in the supplementary materials. Protein expression levels were log_2_-transformed and normalized. Proteins with more than 50% missing values were excluded from the analysis. Missing values were imputed using multiple imputation by chained equations (MICE). Each protein was assigned to one of five immune types (adaptive, innate, both, unspecified, or non-immune), based on whether its Gene Ontology (GO) [17] biological process annotations included any child term of the adaptive immune response (GO:0002250), innate immune response (GO:0045087), or the broader immune system process (GO:0002376).

### Low-pass whole genome sequencing

Genotyping was performed by BGI (formally Beijing Genomics Institute) using low-pass whole genome sequencing (WGS) and imputed with Gencove’s ImputeSeq [18], with the same blood samples collected for proteomics. Single Nucleotide Polymorphisms (SNPs) were filtered by excluding the X chromosome, having a minor allele frequency (MAF) < 0.05, failing Hardy-Weinberg equilibrium (HWE) at *p* < 1 × 10⁻⁶, and removing duplicated Reference SNP cluster IDs (rsid).

### Statistical analysis

#### Differentially expressed protein analysis

Four models were fit to compare protein expression levels between cotton workers and endotoxin-free silk workers. Each model assessed the association using either raw or MICE-imputed data. Endotoxin exposure was evaluated either as a binary variable (cotton vs. silk) or as a continuous variable (natural log-transformed cumulative endotoxin concentration). All modelsadjusted for sex (male, female), age (years), smoking pack-years, body mass index (BMI; kg/m^2^), and the first three principal components (PCs) of all protein expressions. Differentially expressed protein (DEP) candidates were determined when proteins were significant in at least two of the four regression models. Bonferroni-corrected p-values < 0.05 were applied to control for multiple testing. To further evaluate whether immune-related proteins were collectively significant (*p* < 0.05), binomial enrichment tests were conducted separately for innate and adaptive immune proteins.

#### Correlation network analysis

A global protein–protein correlation network was first constructed using Prim’s maximum spanning tree (MST) [19] to characterize overall protein correlation patterns among all participants. Occupation-stratified networks of identified DEPs were then constructed to compare differences between cotton and silk workers. To further understand how immune-related proteins interacted within each occupation, immune-specific subnetworks were extracted and analyzed. All networks were evaluated both visually and quantitatively using topological metrics, including network diameter, radius, and heterogeneity. More flexible network structures were generated using Triangulated Maximally Filtered Graph (TMFG) [20] as sensitivity analyses.

#### Functional enrichment

Enrichment analysis was performed using gene annotation from STRING [21]. A signal score, defined as the harmonic mean of the false discovery rate (FDR) and the proportion of observed genes to background genes, was used to quantify the strength of enrichment. For each biological term or pathway, the proportion of downregulated genes was determined, with the statistical significance evaluated by the Wilcoxon signed-rank test.

#### Causal mediation analysis

High-Dimensional Mediation Testing (HDMT) [22] and the modified Divide-Aggregate Composite-null Test (DACT) [23, 24] were applied to identify proteins that mediated the association between endotoxin exposure and lung function decline. Both mediation and outcome models were adjusted for sex, age, smoking pack-years, height, BMI, and the first three PCs of overall protein expression. Bonferroni-corrected p-values < 0.05 were applied to control for multiple testing. The significance of the proportion of the association mediated by each significant protein mediator was estimated using bootstrapping (*n* = 1000). Binomial enrichment tests were conducted to determine whether innate and adaptive immune proteins were significantly (*p* < 0.05) mediated endotoxin-related lung function decline.

#### Mendelian randomization

Significant protein mediators identified through causal mediation analysis were integrated with low-pass GWAS data to identify SNPs associated with these protein mediators using an additive model. To reduce horizontal pleiotropy, SNPs were filtered by linkage disequilibrium (LD) pruning, retaining only SNPs with r² < 0.5 within 50 base pairs. Candidate SNPs were then used as instrumental variables to assess the causal effect of protein expression on lung function decline. Inverse Variance Weighting (IVW) was employed as the primary Mendelian Randomization (MR) method with other commonly used MR methods as sensitivity analyses. SNPs with p-values < 5 × 10⁻⁶ for both the SNP–protein association and the SNP–lung function mediation test using DACT were selected for MR analysis. In addition, the effects of protein expression on lung function decline were estimated directly using adjusted ordinary least squares (OLS) regression models for comparison.

## Results

### Characteristics of participants

**Fig. 1:**
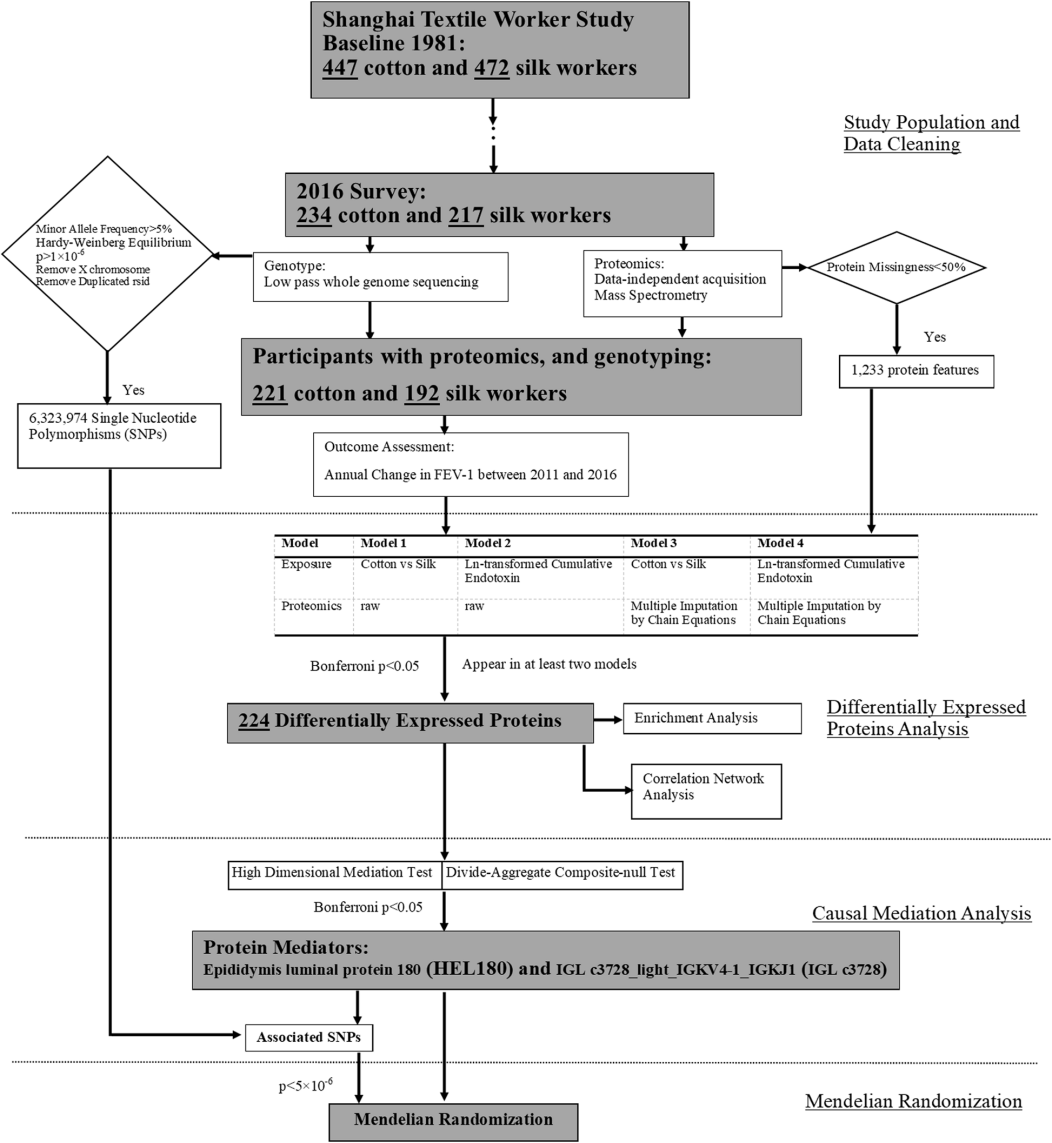
Overall study design and flow chart of statistical analysis

The participant selection and statistical analysis steps are listed in (Fig. 1). The characteristics of the study population are shown in Table 1. Baseline characteristics of cotton and silk workers were similar, with no significant differences in age, sex, and smoking status distribution (*p* > 0.05). Cotton workers had sharper annual declines in FEV-1 compared to silk workers between 2011 and 2016 (−25 vs. −23 ml/year, *p* = 0.02). Cotton workers lost more in FEV-1 over the 35-year follow-up compared to silk workers (900 ml or 30% vs. 800 ml or 27.6%).

**Table 1:** Characteristics of participants with proteomics assessed in the Shanghai Textile Worker Study (*N* = 413)

	Cotton	Silk
	*N* = 221	*N* = 192
Sex		
Male	73 (33%)	57 (30%)
Female	148 (67%)	135 (70%)
Age in 2016 (year), Mean (SD)	70 (± 8.7)	70 (± 8.6)
Cumulative endotoxin concentration (1000 EU/m^3^-year), Mean (SD)	46 (± 40)	0 (± 0)
Smoking status in 2016		
Former	32 (14%)	24 (12%)
Current	34 (15%)	23 (12%)
Never	155 (70%)	145 (76%)
Total smoking pack-year in 2016 (pack-year), Mean (SD)	9.9 (± 22)	6.6 (± 15)
FEV-1 in 2011 (ml), Mean (SD)	2328 (± 619)	2319 (± 571)
FVC in 2011 (ml), Mean (SD)	3023 (± 774)	3002(± 741)
Percent predicted* FEV-1 in 2011 (%),Mean (SD)	94.8 (± 16.2)	97.8 (± 15.7)
Percent Predicted FVC in 2011 (%), Mean (SD)	82.6 (± 13.2)	85.0 (± 12.5)
FEV-1 in 2016 (ml), Mean (SD)	2100 (± 590)	2100 (± 550)
FVC − 1 2016 (ml), Mean (SD)	2757 (± 738)	2772 (± 722)
Percent predicted FEV-1 in 2016 (%), Mean (SD)	91.3 (± 16.7)	93.8 (± 15.6)
Percent predicted FVC in 2016 (%), Mean (SD)	74.9 (± 13.2)	78.1 (± 12.0)
Annual change in FEV-1 between 2011 and 2016 (ml/year), Mean (SD)	−25 (± 11)	−23 (± 9.6)
Annual change in FVC between 2011 and 2016 (ml/year), Mean (SD)	−42.9 (± 41)	−36 (± 43)

Definition of *abbreviations EU* endotoxin unit, *FEV-1* forced expiratory volume in 1 s, *SD *standard deviation, *FVC* Force Vital Capacity

Data are expressed as mean (± SD) for continuous variables and n (%) for categorical variables.*Percent predicted FEV-1 and FVC were calculated from Global Lung Initiative race-neutral formula

### DEPs between endotoxin-exposed cotton and endotoxin-free silk workers

A total of 2,962 proteins were quantified. Out of the 1,233 protein features with less than 50% missing data, 592 proteins were annotated as immune-related proteins (95 innate and 144 adaptive immune proteins). Differential expression analysis revealed that 224 proteins were differentially expressed between endotoxin-exposed cotton and endotoxin-free silk workers. Of these 224 Bonferroni-significant DEPs, 96 proteins were immune-related proteins (20 innate and 40 adaptive immune proteins). Both adaptive (binomial *p* = 9.63 × 10⁻⁹⁷) and innate (binomial *p* = 6.59 × 10⁻³³) immune protein expressions were collectively disrupted by endotoxin, as indicated by highly enriched proportions, suggesting broad immune downregulation with chronic endotoxin exposure. Adaptive and innate proteins were significant (binomial *p* < 0.05) in sub-cohort analyses of female, smokers, non-smokers, and cotton workers only. (Supplement Table S1)

### Correlation networks analysis

In the MST constructed for all workers (Supplement Figure S1), Profilin-1 (UniProt ID: P07737; betweenness centrality = 0.69) was identified as the central node. Elongation factor G, mitochondrial (UniProt ID: Q96RP9) exhibited the highest degree within the MST, particularly connecting with a large number of proteins with limited annotation.

**Fig. 2:**
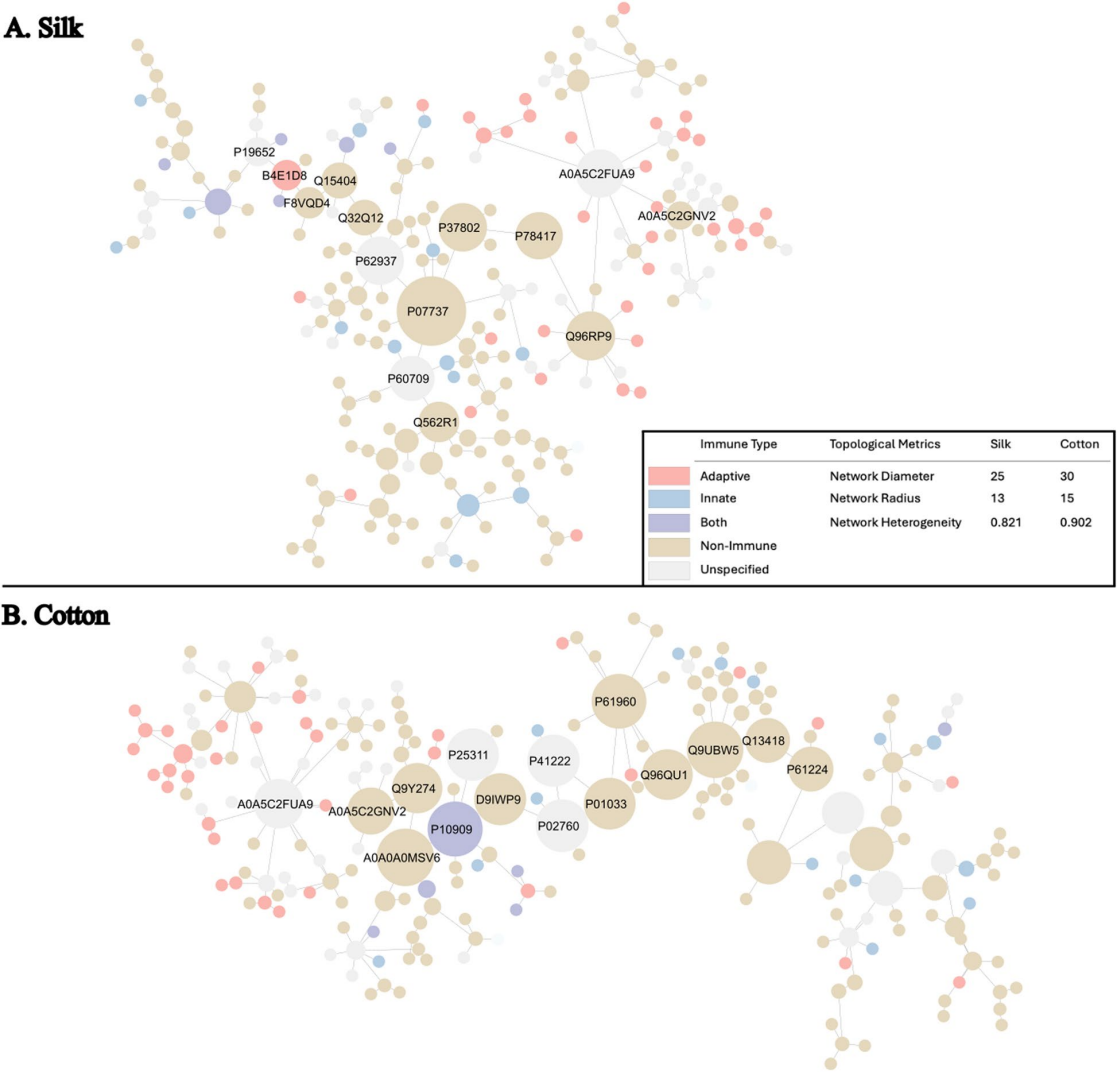
Protein–protein correlation networks of differentially expressed proteins associated with occupational endotoxin exposure, stratified by occupation. **A** Silk workers; **B** cotton workers. Networks were constructed using Prim’s maximum spanning tree. Only the top 15 proteins ranked by betweenness centrality are labeled

When comparing the stratified networks by occupation, the network of cotton workers (Fig. 2B) displayed a more complex and heterogeneous structure than that of the silk workers (Fig. 2A). Cotton workers compared to silk workers showed higher network diameter (30 vs. 25), network radius (15 vs. 13), and network heterogeneity (0.90 vs. 0.82). The more heterogeneous network structure in cotton workers was also observed in immune subnetworks and sensitivity graphs (Supplemental Table S2 and Supplemental Figure S2). These topological differences suggest that endotoxin exposure may disrupt protein expression and alter the protein-protein correlation structure, particularly in immune-related proteins by increasing variability and forming more extreme network hubs.

### Functional enrichment of DEPs

For cellular components, we observed significant differences in protein expression levels, particularly among vesicle- and extracellular region–related components. Cotton workers exhibited significantly altered protein expression in vesicles (GO:0031982, *n* = 94, FDR = 1.49 × 10⁻³², signal = 1.48) and extracellular spaces (GO:0005615, *n* = 99, FDR = 2.37 × 10⁻⁴⁴, signal = 1.92) compared to silk workers, especially within blood microparticles (GO:0072562, *n* = 18, FDR = 3.54 × 10⁻¹⁶, signal = 3.18). Among vesicle-related components, both intracellular vesicles (GO:0097708, *n* = 49, FDR = 2.10 × 10⁻¹⁰, signal = 0.96) and extracellular exosomes (GO:0070062, *n* = 80, FDR = 2.04 × 10⁻³⁹, signal = 2.26) were significantly enriched. The most enriched component within intracellular vesicles was the secretory granule lumen (GO:0034774, *n* = 27, FDR = 1.23 × 10⁻¹⁸, signal = 2.82).

At the level of molecular function, proteins associated with hydrolase activity (GO:0016787) were significantly downregulated in cotton workers. This included serine-type endopeptidase inhibitor activity (GO:0004867, *n* = 15, FDR = 1.95 × 10⁻⁸, signal = 1.2) and GTPase activity (GO:0003924, *n* = 11, FDR = 0.0055, signal = 0.51). Additionally, proteins involved in binding functions such as cadherin binding (GO:0045296, *n* = 13, FDR = 0.00003, signal = 0.75) and GTP binding (GO:0005525, *n* = 12, FDR = 0.0055, signal = 0.51) were significantly less expressed in the cotton group.

In terms of biological processes, 135 distinct processes showed differential enrichment between cotton and silk workers. The processes with the highest signal values included regulation of endopeptidase activity (GO:0052548, *n* = 20, FDR = 1.20 × 10⁻⁷, signal = 1.28), inflammatory response (GO:0006954, *n* = 20, FDR = 2.88 × 10⁻⁵, signal = 1.01), and negative regulation of hydrolase activity (GO:0051346, *n* = 16, FDR = 8.48 × 10⁻⁶, signal = 1.01). Regulation of the immune system process was also significantly enriched (GO:0002682, *n* = 29, FDR = 8.95 × 10⁻^5^, signal = 0.65).

KEGG pathway analysis revealed significant enrichment in both the complement and coagulation cascades (KEGG: hsa04610, *n* = 9, FDR = 3.94 × 10⁻⁶, signal = 1.30) and the chemokine signaling pathway (KEGG: hsa04062, *n* = 11, FDR = 8.25 × 10⁻⁵, signal = 1.04). The top three enriched Reactome pathways were platelet activation, signaling and aggregation (HSA-76002, *n* = 31, FDR = 5.41 × 10⁻²⁵, signal = 3.90), platelet degranulation (HSA-114608, *n* = 21, FDR = 3.29 × 10⁻¹⁹, signal = 3.71), and hemostasis (HSA-109582, *n* = 36, FDR = 4.09 × 10⁻²⁰, signal = 2.46).

On average, protein expression levels were significantly (Wilcoxon signed-rank test *p* < 0.05) lower in cotton workers compared to silk workers across all biological terms and pathways, with a minimum proportion of downregulated genes of 78.57%. Detailed enrichment results are shown in Supplementary Table S3.

### Protein mediators of endotoxin-related lung function change

Causal mediation analysis was conducted to further identify proteins that significantly mediate the effect of endotoxin exposure on lung function decline among the noteworthy DEPs. Among the 224 endotoxin-associated DEPs, 18 immune proteins showed significant (HDMT *p* < 0.05) mediation of the endotoxin–lung function decline relationship (binomial *p* = 3.65 × 10⁻⁷). Enrichment was driven by adaptive immune proteins (*n* = 12, binomial *p* = 1.21 × 10⁻⁶), whereas innate immune proteins showed no enrichment (*n* = 1, binomial *p* = 0.64). These results indicate that adaptive immune pathways are the predominant mediators linking endotoxin exposure to lung function decline, while innate components play a lesser role.

In addition, two immunoglobulin (Ig) domain proteins, Epididymis luminal protein 180 (UniProt ID: B6EDE2, HEL180) and IGL c3728_light_IGKV4-1_IGKJ1 (UniProt ID: A0A5C2G374, IGL c3728), were identified as significant mediators (Bonferroni *p* < 0.05) in models using both raw and imputed protein expression and using both continuous and binary exposure terms. Results were significant in both HDMT and modified-DACT testing (Table 2). After 1,000 bootstrapping iterations, the proportion mediated was estimated at 46.4% (*p* = 0.04) for HEL180 and 46.9% (*p* = 0.05) for IGL c3728. Mediation effects of both IGL c3728 and HEL180 were significant (HDMT *p* < 0.05) in sex-stratified analysis.

**Table 2:** Protein mediators of endotoxin-related lung function changes and summary of mediation analysis results

Protein Names	IGL c3728_light_IGKV4-1_IGKJ1	Epididymis luminal protein 180
Protein ID	A0A5C2G374	B6EDE2
*P*-value of HDMT	9.41 × 10^− 6^	6.83 × 10^− 6^
*P*-value of Modified DACT	1.42 × 10^− 5^	8.71 × 10^− 6^
Average causal mediation effect (95% CI)	−0.73 (−1.29, −0.29)	−0.717 (−1.21, −0.31)
Average direct effect (95% CI)	−0.82 (−2.55, 0.69)	−0.827 (−2.63, 0.69)
Total effect (95% CI)	−1.54 (−3.31, −0.03)	−1.544 (−3.27, −0.06)
Proportion mediated (p-value)	46.9% (0.05)	46.4% (0.04)

Definition of *abbreviations CI *Confidence Interval, *DACT* divide-aggregate composite-null test, *HDMT * high dimensional mediation analysis

### Effect of protein expression on lung function change

We analyzed a total of 6,323,974 SNPs in the mediation analysis to evaluate how significant protein mediators affect lung function change, adjusted for endotoxin exposure. Fourteen SNPs were associated with HEL180 expression (*p* < 5 × 10⁻⁶), and another fourteen SNPs were associated with IGL c3728 expression. The SNP lists were identical between models using binary and continuous endotoxin exposure terms. Details of the associated SNPs are provided in Supplementary Table S4.

**Fig. 3:**
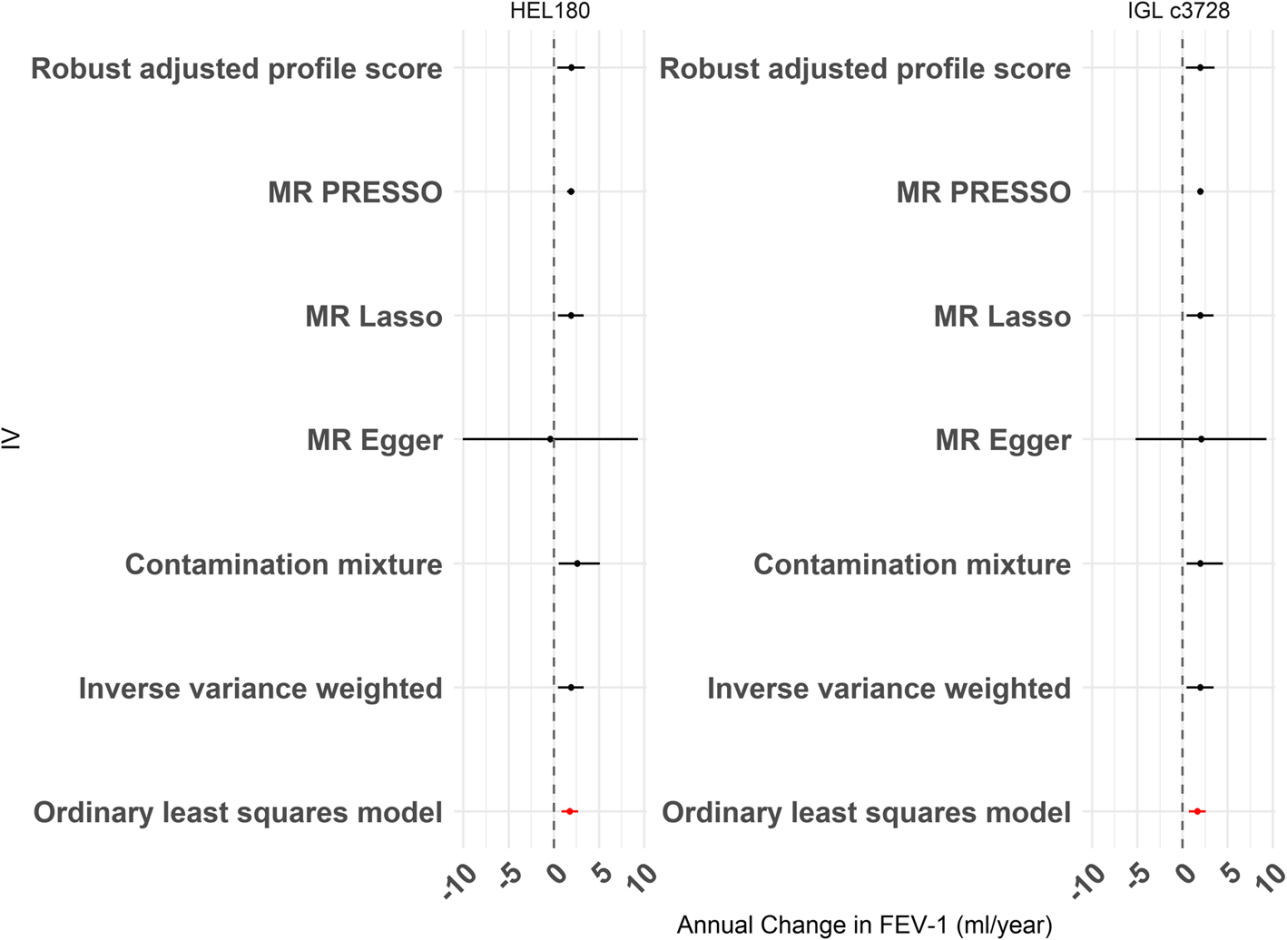
Forest plot of overall estimates for the effect of a two-fold increase in protein mediator expression on lung function change, based on Mendelian Randomization (MR) analyses and adjusted ordinary least squares regression. MR methods include robust adjusted profile score, MR-PRESSO, MR-Lasso, MR-Egger, contamination mixture, and inverse-variance weighted approaches

A forest plot illustrating the estimated effects of HEL180 and IGL c3728 on lung function change is shown in (Fig. 3). In general, protein expressions of HEL180 and IGL c3728 demonstrated a positive association with lung function change. Using IVW, a two-fold decrease in HEL180 expression was associated with a 1.92 ml/year (95% CI: [0.79, 3.02]) decline in FEV-1. For IGL c3728, the decline was 2.35 ml/year (95% CI: [1.68, 3.02]). Finally, in OLS models, every two-fold decrease in HEL180 expression was associated with a 1.79 ml/year (95% CI: [0.86, 2.72]) decline in FEV-1. A similar decline in FEV-1 was observed for every two-fold decrease in IGL c3728 expression (1.64 ml/year, 95% CI: [0.71, 2.57]). Estimates from OLS models were directionally consistent with MR analyses.

## Discussion

We leveraged advanced proteomic profiling, genomics, and causal methods to understand the molecular changes and biological pathways underlying endotoxin-related lung function decline. We identified 224 differentially expressed protein features between endotoxin-exposed cotton workers and endotoxin-free silk workers. Most of these proteomic features were related to inflammatory responses and immune pathways. Among these noteworthy signals, two significant Ig protein features were found to significantly mediate endotoxin-related lung function change: HEL180 and IGL c3728, suggesting endotoxin potentially affects lung function decline through the adaptive immune response.

Our correlation network and enrichment analyses revealed that chronic endotoxin exposure may disrupt both innate and adaptive immunity, leading to impairment of immune and inflammatory regulation. Most of the 224 DEPs between endotoxin-exposed and control textile workers were associated with vesicle-related cellular components. Vesicles, including extracellular vesicles (EVs), are essential for transferring molecules between cells, including immune cells. We also observed downregulation of hydrolase activity in response to endotoxin exposure. Hydrolases are often packaged within vesicles such as lysosomes or secretory granules. Downregulation of hydrolase activity may lead to tissue damage and persistent inflammation [25]. In addition, 78–100% of proteins were downregulated in key inflammatory and immune pathways such as the complement cascade, chemokine signaling, and platelet activation, suggesting broad suppression of immune responses. Collectively, our findings provide evidence supporting endotoxin tolerance in chronically exposed workers, where innate and adaptive immune responses are possibly reprogrammed following repeated exposure to LPS. Such alterations ultimately impair host defense and hemostatic functions that are critical for maintaining lung health [26].

Through mediation analysis, we identified HEL180 and IGL c3728 as significant mediators of the effect of endotoxin exposure on lung function. The protein expression of these two mediators was moderately correlated (*r* = 0.40). Both proteins belong to the Ig domain family. Ig domain proteins play key roles in adaptive immune responses, including antigen recognition and immune signaling [27]. In the respiratory system, local antibody production is critical for the neutralization of inhaled pathogens and mucociliary clearance. While HEL180 was primarily characterized as an epithelial secretory protein in reproductive tissue, the identification of HEL180 as mediator protein may reflect a research focus on tissue-specific rather than true sex-restricted expression. Many epithelial secretory proteins initially identified in the epididymis have since been found to be expressed in diverse mucosal tissues. For example Human Epididymis Protein 4 (HE4) is expressed across multiple epithelial tissues, where it is involved in innate immunity at mucosal barriers [28] and has been associated with decrease lung function in fibrotic lung diseases [29] Although direct functional relationships between HEL180 and other epithelial proteins remain unclear, their common expression in epithelial tissues suggests that they may serve similar biological roles, potentially including barrier protection and immune defense at mucosal surfaces. In contrast, downregulation of IGL c3728, an Ig light chain variant, directly indicates reduced antibody production in endotoxin-exposed workers. Importantly, adaptive immune proteins rather than innate immune proteins were collectively significant mediators of endotoxin-related lung function decline. These findings align with prior studies showing that impaired adaptive immunity, such as T-cell or B-cell deficiency, is associated with worsened lung damage and delayed recovery [30, 31]. Additional studies in human populations are needed to better understand the role of adaptive immune proteins as mediators of lung function loss.

Our study has notable strengths. First, it is the largest occupational study of textile workers with the longest follow-up period to date. Our findings help expand the understanding of endotoxin-related lung function changes to the molecular level. Our exposure assessment of endotoxin is robust. Although we used area sampling for cotton dust, a prior validation study showed a high correlation between area and personal sampling based on job titles [32]. Additionally, our analytical approach addresses a complex research question using multi-omics. Using proteomics profiling of cotton and silk workers, we identified protein mediators of endotoxin-related lung function decline. Sequentially, we applied low-pass WGS to mitigate the risk of unmeasured confounding, we employed MR, which further supports the influence of HEL180 and IGL c3728 on lung function change found in the OLS estimates. We applied several MR methods to minimize potential bias from horizontal pleiotropy and found similar results as the gold standard IVW.

Our study has several limitations. First, due to the nature of an occupational longitudinal study design, selection biases, such as the healthy worker survival effect and loss to follow-up, are difficult to avoid. In this follow-up study, we observed that the 413 participants who had proteomics and genotyping assessed had better lung functions and fewer respiratory symptoms than those who did not participate at baseline. Such a difference could bias the effect estimate toward the null. Second, although we implemented a robust exposure assessment, all participants were retired and had ceased occupational exposure to endotoxin by the time of our most recent survey in 2016. Proteomic measurements were only taken at a single cross-sectional time point. Our previous research suggested that some effects of endotoxin exposure on lung damage may be partially reversible [11]. While our focus was on the long-term consequences of exposure, by the time proteomic data were collected, some adverse health effects, whether related to lung function or protein expression, may have already partially recovered. Consequently, our estimates and findings may be conservative. Future studies could explore longitudinal changes in protein expression through measurements at two or more time points. Moreover, due to the uniqueness of our cohort, replicating our results poses a challenge, particularly with long-term repeated measurement of endotoxin. This may limit the generalizability of our results.

Despite decades of research, there is currently no consensus on occupational exposure limits for endotoxins [33]. In Europe, the Dutch Expert Committee on Occupational Safety (DECOS) initially recommended a limit of 50 EU/m³ in 1998, which was raised to 200 EU/m³ in 2005 and later reduced to 90 EU/m³ in 2010 [34]. In the U.S., although the Occupational Safety and Health Administration has established permissible exposure limits (PELs) for cotton dust, there are no PEL standards for endotoxin exposure. In our cohort, most cotton textile workers worked in environments where endotoxin levels exceeded the 8-hour time-weighted average of 50 EU/m³ until retirement or cessation of exposure. Our results show a range of immunologic changes and respiratory effects in response to occupational exposure to airborne endotoxins. To protect the health of workers exposed to endotoxin, further research and regulatory actions are needed to establish clear exposure limits in workplaces. 

## Conclusion

This study provides biological insights into mechanisms underlying endotoxin-related lung function decline. Our findings suggest chronic endotoxin exposure impairs broad immunity and that through adaptive immune proteins, endotoxin reduces lung function. We also identified two potential adaptive immune protein mediators. Our findings also emphasize the importance of workplace interventions to reduce or eliminate endotoxin exposure and promote respiratory health among susceptible populations in LMICs. However, given the caveats of our study, caution is recommended when interpreting the findings.

## Supplementary Information

Supplementary Material 1.

## Data Availability

Data used in this study are available upon reasonable request and subject to approval by the Institutional Review Board of the Harvard T.H. Chan School of Public Health. A data use agreement must be in place prior to data transfer. For inquiries, please contact Dr. David Christiani at dchris@hsph.harvard.edu. The detailed statistical analysis plan is included in the supplemental materials. Programming code used in the study is available upon request.
