# Supplementary material for: Proteomic profiling reveals immunologic mediators of endotoxin-related lung function decline: a longitudinal prospective study in textile workers
Source: Respir Res. 2026 Apr 17;27:234. doi: 10.1186/s12931-026-03669-4 (PMC13262221; doi:10.1186/s12931-026-03669-4)
Supplement: Supplementary file 1 — Supplementary Material 1. [file 12931_2026_3669_MOESM1_ESM.docx]

**1. Supplementary Methods**

**1.1 Study Population:**

The Shanghai Textile Worker Study is a longitudinal occupational cohort study initiated in 1981, consisting of 447 cotton workers and 472 silk workers in Shanghai, China. Follow-up surveys were conducted in 1986, 1992, 1996, 2001, 2006, 2011, and 2016^1-6^. Endotoxin-free silk workers were recruited as the reference group. Both cotton and silk textile workers lived in the same neighborhood and shared similar demographic characteristics and environmental exposures except for occupational exposure to cotton dust and endotoxin. Workers might leave or switch jobs within mills, but they never switched to other mills, therefore silk workers were not exposed to occupational cotton dust and endotoxin during study period. At each survey, work history, respiratory symptoms, smoking history, and other information were collected using a modified version of the standardized respiratory symptom questionnaire from the ATS^7^. The questionnaires were administered by trained local interviewers and were translated into English. All participants were provided with informed consent. Ethical approval was obtained from the Institutional Review Boards of the Harvard School of Public Health and Shanghai Putuo District People's Hospital.

**1.2 Exposure Assessment:**

Area full-shift samples of cotton dust were collected on-site using vertical elutriators during each survey prior to the mill closures in 2001. Multiple area samples from six different work areas were collected in two cotton mills, with sampling time ranging from 3 to 8 hours. Endotoxin concentrations were analyzed in the National Institute of Occupational Safety and Health (NIOSH) lab using Limulus Amebocyte Lysate (Pyrostat-50). The dust and endotoxin concentrations at the silk mill were lower than detection levels.

The primary exposure of interest was cumulative endotoxin concentration (EU/m³), which was approximated based on participants' job titles and detailed work history. Endotoxin concentration at each work area was averaged by geometric means. Each participant at each survey was assigned by his or her work area and time worked since the previous survey. Although we used area samples to approximate personal exposure to endotoxin, validation studies indicated a high correlation between area and personal exposure^8^. In this study, endotoxin exposure was assessed both as a binary variable based on occupation (cotton vs silk) and as the natural logarithm (ln)-transformed cumulative endotoxin concentration. The cumulative endotoxin values were ln-transformed to reduce skewness in the distribution.

The endotoxin concentration of cotton workers decreased over time. During the early 1990s, the cotton mills began blending synthetic fibers with cotton and reduced pure cotton yarn production. This production line change significantly reduced cotton dust in the workplace. In addition, most participants started to retire and cease textile work in the late 1990s, resulting in no occupational exposure to endotoxin.

**1.3 Outcome Assessment:**

Forced expiratory volume in 1 second (FEV-1) was measured following the spirometry technical standards of the American Thoracic Society (ATS)^9^. Spirometry curves were measured using a calibrated 8-liter water-sealed field spirometer (W E Collins, Braintree, Massachusetts, USA). FEV-1 readings were determined manually by trained experts. At least three measurements were recorded. The highest FEV-1 measurement from each test was used for analysis. All FEV-1 values were adjusted for Body Temperature and Pressure, Saturated (BTPS). The primary outcome was the annual change in FEV-1 between the 2011 and 2016 surveys (ml/year), with positive values indicating an improvement in lung function and negative values indicating a decline.

**1.4 Proteomics**

Serum samples were collected from 221 cotton workers and 216 silk workers in 2016 for proteomic analysis. Protein quantification was performed using data-independent acquisition mass spectrometry (DIA-MS) on a high-performance liquid chromatography coupled with a Q Exactive HF-X hybrid quadrupole-Orbitrap mass spectrometer system (Thermo Fisher Scientific, San Jose, CA) by BGI Genomics (Shenzhen, China). Details of sample preparation can be found in our previous study^10^. Protein expression levels were log_2_ transformed and normalized. Proteins with more than 50% missing values were excluded from the analysis. Missing values were imputed using Multiple Imputation by Chained Equations^11^ (MICE). Prior to imputation, least absolute shrinkage and selection operator (LASSO) regression was performed for variable selection. Bayesian linear regression was used for the imputation model, with 40 imputations and a maximum of 30 iterations per imputation.

Each protein was assigned to one of five immune types (adaptive, innate, both, unspecified, or non-immune), based on whether its Gene Ontology (GO)^12^ biological process annotations included any child term of the adaptive immune response (GO:0002250), innate immune response (GO:0045087), or the broader immune system process (GO:0002376).

**1.5 Low-pass Whole Genome Sequencing (WGS)**

Genotyping was performed using BGI’s low-pass whole-genome sequencing and imputed with Gencove’s ImputeSeq^13^ with the same blood samples collected for proteomics. Low-pass WGS increases statistical power compared to genotyping arrays and is significantly less biased towards European populations. This means increased power for a fixed study budget and no customizations are required for non-European populations. Gencove’s highly accurate ImputeSeq calls variants across the whole genome with an average imputation concordance with deep WGS of 99% for 1x coverage and 99.5% for 4x. Imputation accuracy across all allele frequencies is higher from low-pass sequencing relative to commonly used genotyping arrays. Single Nucleotide Polymorphisms (SNPs) were filtered by excluding the X chromosome, with a minor allele frequency (MAF) < 0.05, failing Hardy-Weinberg equilibrium (HWE) at p < 1×10⁻⁶, or removing duplicated Reference SNP cluster IDs (rsid).

**1.6 Statistical Analysis**

**1.6.1 Differentially Expressed Protein Analysis**

DEP analysis was conducted to compare protein expression levels between cotton workers and endotoxin-free silk workers. Log_2_-transformed protein expression levels were regressed on endotoxin exposure using linear regression models, adjusting for sex (male, female), age (year), smoking pack-years, body mass index (BMI; kg/m^2^), and the first three principal components (PCs) of overall protein expression. DEP candidates were identified using a majority voting approach across four regression models, each assessing the association between endotoxin exposure and protein expression using either raw or MICE-imputed data. Endotoxin exposure was evaluated both as a binary variable (cotton vs silk) and as a continuous variable (log-transformed cumulative endotoxin concentration). Bonferroni-corrected p-values < 0.05 were applied to control for multiple testing. To further evaluate whether immune-related proteins were collectively significant (p<0.05), binomial enrichment tests were conducted separately for innate and adaptive immune proteins. DEP analysis and binomial enrichment tests were performed in sub-cohort of female, male, smoker, non-smoker, and cotton workers as sensitivity analysis.

**1.6.2 Network Correlation Analysis**

A protein-protein correlation network was constructed across all participants using Prim’s maximum spanning tree (MST) algorithm^14^, implemented via CyFinder in Cytoscape^15^. Node sizes represented betweenness centrality, and edge weights reflected pairwise correlation values between proteins. Nodes were colored according to K-means clustering assignments for well-annotated proteins, and less-annotated proteins were included to visualize their relationships with nearby well-annotated proteins. Additionally, occupation-stratified networks were generated separately for cotton and silk workers to compare protein-protein correlation patterns. These networks were evaluated both visually and quantitatively using topological metrics, including network diameter, network radius, and network heterogeneity. To further understand how immune-related proteins interacted within each occupation, immune-specific subnetworks were extracted and analyzed. All networks were evaluated both visually and quantitatively using topological metrics, including network diameter, radius, and heterogeneity. More flexible network structures were generated using Triangulated Maximally Filtered Graph (TMFG)^16^ as sensitivity analysis.

**1.6.3 Function Enrichment**

Enrichment analysis was performed using gene annotation resources including GO, KEGG^17^, and Reactome^18^, accessed via the STRING^19^ using an over-representation approach. A signal score, defined as the harmonic mean of the false discovery rate (FDR) and the proportion of gene features present in the background set, was used to quantify the strength of enrichment. For each biological term or pathway, the proportion of downregulated genes was determined, and the Wilcoxon signed-rank test was used to evaluate the statistical significance of the observed gene expression changes. Enrichment scores and p-values were computed using protein-level beta values to assess overrepresentation among proteins ranked by effect size.

**1.6.4 Causal Mediation Analysis**

We applied a causal mediation analysis framework based on the definitions of the Natural Direct Effect (NDE) and Natural Indirect Effect (NIE)^20^. To identify proteins that mediate the association between endotoxin exposure and lung function decline, we applied High-Dimensional Mediation Testing^21^ (HDMT) and the Modified Divide-Aggregate Composite-null Test^22,23^ (DACT). Both mediation and outcome models were adjusted for sex, age, smoking pack-years, height, BMI, and the first three principal components (PC1–PC3) of the overall protein expression. Standard errors and p-values of the proportion of the association mediated by each protein were estimated using bootstrap (n=1000). Binomial enrichment tests were conducted to determine whether innate and adaptive immune proteins were significantly (p<0.05) mediated endotoxin-related lung function decline. SNPs associated with the protein mediators of endotoxin-related lung function decline were further identified with low-pass GWAS data.

HDMT and DACT differ from traditional mediation testing methods such as Sobel’s and MaxP tests by decomposing the null hypothesis of the indirect effect into three sub-hypotheses: (1) exposure affects the mediator (β ≠ 0) but the mediator does not affect the outcome (γ = 0), (2) exposure does not affect the mediator (β = 0) but the mediator affects the outcome (γ ≠ 0), and (3) both β and γ are zeros. In practice, because mediation analysis was performed following DEP analysis, the association between endotoxin exposure and DEP expression (β ≠ 0) was already established. Therefore, the mediation testing largely approximates testing the association between protein expression and lung function change (γ = 0). Similarly, for the mediation analysis of SNP–protein–lung function change, the mediation test approximates a GWAS of protein expression (testing β = 0).

**1.6.5 Mendelian Randomization**

Significant protein mediators identified through causal mediation analysis were integrated with low-pass GWAS data to identify SNPs associated with the protein mediators of endotoxin-related lung function decline using an additive model. To reduce horizontal pleiotropy, SNPs were filtered by linkage disequilibrium (LD) pruning, retaining only SNPs with r² < 0.5. Candidate SNPs were then used as instrumental variables to assess the causal effect of protein expression on lung function decline using Mendelian Randomization (MR). Inverse Variance Weighting (IVW) was employed as the primary MR method, with MR-RAPS^24^, MR-PRESSO^25^, MR-LASSO^26^, contamination mixture^27^, and MR Egger^28^ used as sensitivity analyses to account for horizontal pleiotropy. SNPs with p-values < 5×10⁻⁶ for both the SNP–protein association^29^ and the SNP–lung function mediation test using DACT were selected for MR analysis. In addition, the effects of protein expression on lung function decline were estimated directly using adjusted ordinary least squares regression models for comparison.

**2. Supplementary Tables**

**Supplemental Table S1. Details of endotoxin-related differentially expressed proteins.**
Z-scores of sensitivity are color-coded to indicate direction and magnitude (blue, negative; white, 0; red, positive), enabling consistency checks.

| Protein Information | |  |  |  | Sensitivity Analysis Z score in sub-cohort | | | |  |
| --- | --- | --- | --- | --- | --- | --- | --- | --- | --- |
| Protein ID | Protein Name(s) | Gene(s) | Immunity Type | P value | female | male | Non-smoker | smoker | cotton |
| Q6P2Q9 | Pre-mRNA-processing-splicing factor 8 (220 kDa U5 snRNP-specific protein) (PRP8 homolog) (Splicing factor Prp8) (p220) | PRPF8 PRPC8 | non-immune | 4.21E-25 | -9.83 | -5.22 | -10.00 | -4.09 | -1.53 |
| A0A0A0MSV6 | Complement C1q B chain | C1QB | non-immune | 2.69E-17 | -8.66 | -3.24 | -8.97 | -2.06 | -1.97 |
| P08185 | Corticosteroid-binding globulin (CBG) (Serpin A6) (Transcortin) | SERPINA6 CBG | non-immune | 3.40E-17 | -7.80 | -3.92 | -7.74 | -3.82 | -0.04 |
| Q9UBV8 | Peflin (PEF protein with a long N-terminal hydrophobic domain) (Penta-EF hand domain-containing protein 1) | PEF1 ABP32 UNQ1845/PRO3573 | non-immune | 4.58E-15 | -8.30 | -3.49 | -8.25 | -3.36 | -1.15 |
| A0A5C2FWP7 | IGL c606_light_IGKV1-12_IGKJ1 |  | unspecified | 1.47E-14 | -7.31 | -3.46 | -6.41 | -4.74 | -0.23 |
| Q6MZL2 | Uncharacterized protein DKFZp686M0562 | DKFZp686M0562 | innate | 4.58E-14 | -7.97 | -3.44 | -7.82 | -3.23 | -1.09 |
| Q8TAL5 | Uncharacterized protein C9orf43 | C9orf43 | non-immune | 6.17E-14 | -5.91 | -2.05 | -5.81 | -2.36 | 1.50 |
| P02763 | Alpha-1-acid glycoprotein 1 (AGP 1) (Orosomucoid-1) (OMD 1) | ORM1 AGP1 | unspecified | 7.92E-14 | -6.30 | -4.36 | -6.56 | -3.80 | 0.12 |
| A0N5G1 | Rheumatoid factor C6 light chain | V-kappa-1 | non-immune | 1.04E-13 | -6.67 | -4.11 | -6.50 | -4.03 | -0.74 |
| P41222 | Prostaglandin-H2 D-isomerase (EC 5.3.99.2) (Beta-trace protein) (Cerebrin-28) (Glutathione-independent PGD synthase) (Lipocalin-type prostaglandin-D synthase) (L-PGDS) (Prostaglandin-D2 synthase) (PGD2 synthase) (PGDS) (PGDS2) | PTGDS PDS | unspecified | 4.92E-13 | -6.45 | -3.69 | -6.56 | -3.00 | -0.63 |
| Q2L9S7 | Alpha-1-antitrypsin MBrescia variant | AAT | non-immune | 5.41E-13 | -6.79 | -3.31 | -6.48 | -3.60 | -0.77 |
| P19652 | Alpha-1-acid glycoprotein 2 (AGP 2) (Orosomucoid-2) (OMD 2) | ORM2 AGP2 | unspecified | 3.45E-12 | -5.85 | -4.07 | -5.99 | -3.37 | -0.35 |
| P01011 | Alpha-1-antichymotrypsin (ACT) (Cell growth-inhibiting gene 24/25 protein) (Serpin A3) [Cleaved into: Alpha-1-antichymotrypsin His-Pro-less] | SERPINA3 AACT GIG24 GIG25 | non-immune | 7.91E-12 | -5.74 | -4.39 | -6.19 | -3.21 | -0.39 |
| P02656 | Apolipoprotein C-III (Apo-CIII) (ApoC-III) (Apolipoprotein C3) | APOC3 | non-immune | 1.54E-11 | 7.82 | 1.18 | 7.67 | 0.88 | -1.78 |
| P11684 | Uteroglobin (Club cell phospholipid-binding protein) (CCPBP) (Club cells 10 kDa secretory protein) (CC10) (Secretoglobin family 1A member 1) (Urinary protein 1) (UP-1) (UP1) (Urine protein 1) | SCGB1A1 CC10 CCSP UGB | unspecified | 1.99E-11 | 7.34 | 2.25 | 7.65 | 1.99 | 1.28 |
| Q99081 | Transcription factor 12 (TCF-12) (Class B basic helix-loop-helix protein 20) (bHLHb20) (DNA-binding protein HTF4) (E-box-binding protein) (Transcription factor HTF-4) | TCF12 BHLHB20 HEB HTF4 | unspecified | 2.25E-11 | -6.02 | -4.54 | -6.57 | -3.28 | -3.44 |
| P25311 | Zinc-alpha-2-glycoprotein (Zn-alpha-2-GP) (Zn-alpha-2-glycoprotein) | AZGP1 ZAG ZNGP1 | unspecified | 3.08E-11 | -5.77 | -3.73 | -5.71 | -3.27 | -0.32 |
| S6AWD6 | IgG L chain |  | unspecified | 5.45E-11 | -5.62 | -3.50 | -5.73 | -3.37 | 1.55 |
| Q10469 | Alpha-1,6-mannosyl-glycoprotein 2-beta-N-acetylglucosaminyltransferase (EC 2.4.1.143) (Beta-1,2-N-acetylglucosaminyltransferase II) (GlcNAc-T II) (GNT-II) (Mannoside acetylglucosaminyltransferase 2) (N-glycosyl-oligosaccharide-glycoprotein N-acetylglucosaminyltransferase II) | MGAT2 | non-immune | 6.00E-11 | -5.62 | -3.37 | -6.56 | -1.80 | -0.33 |
| A0A5C2GD65 | IGH + IGL c638_light_IGKV3-11_IGKJ5 |  | unspecified | 7.72E-11 | -5.99 | -3.17 | -5.83 | -3.23 | 0.20 |
| A0A5C2GWK8 | IG c98_light_IGKV2D-28_IGKJ1 |  | unspecified | 1.30E-10 | -5.40 | -3.51 | -5.35 | -3.44 | -0.55 |
| A0A087WSY6 | Immunoglobulin kappa variable 3D-15 | IGKV3D-15 | adaptive | 1.36E-10 | -5.65 | -3.44 | -5.28 | -3.58 | 0.43 |
| Q6ZU69 | Protein SPATA31F1 (Protein FAM205A) | SPATA31F1 C9orf144B FAM205A | non-immune | 1.75E-10 | -6.97 | -1.65 | -6.39 | -2.24 | -0.41 |
| P60953 | Cell division control protein 42 homolog (EC 3.6.5.2) (G25K GTP-binding protein) | CDC42 | innate | 3.10E-10 | -5.91 | -2.63 | -5.52 | -2.86 | -1.43 |
| Q9BWU5 | Mutant hemoglobin beta chain | HBB | non-immune | 3.28E-10 | -6.50 | -2.00 | -5.98 | -2.39 | -1.45 |
| P01023 | Alpha-2-macroglobulin (Alpha-2-M) (C3 and PZP-like alpha-2-macroglobulin domain-containing protein 5) | A2M CPAMD5 FWP007 | innate | 3.55E-10 | -4.70 | -4.57 | -5.34 | -2.98 | 0.72 |
| P02776 | Platelet factor 4 (PF-4) (C-X-C motif chemokine 4) (Iroplact) (Oncostatin-A) [Cleaved into: Platelet factor 4, short form (Endothelial cell growth inhibitor)] | PF4 CXCL4 SCYB4 | unspecified | 3.78E-10 | -5.08 | -4.13 | -5.29 | -3.30 | -1.38 |
| Q6VFQ6 | Hemoglobin beta chain | HBB | non-immune | 3.98E-10 | 6.77 | 1.52 | 6.51 | 1.42 | 0.26 |
| A0A5C2GKQ4 | IG c1117_light_IGKV3-11_IGKJ1 |  | unspecified | 4.40E-10 | -5.82 | -2.91 | -5.43 | -3.21 | -0.44 |
| O14793 | Growth/differentiation factor 8 (GDF-8) (Myostatin) | MSTN GDF8 | unspecified | 4.80E-10 | -5.59 | -3.72 | -5.96 | -2.80 | -0.16 |
| P16885 | 1-phosphatidylinositol 4,5-bisphosphate phosphodiesterase gamma-2 (EC 3.1.4.11) (Phosphoinositide phospholipase C-gamma-2) (Phospholipase C-IV) (PLC-IV) (Phospholipase C-gamma-2) (PLC-gamma-2) | PLCG2 | innate | 7.90E-10 | -5.87 | -2.67 | -5.76 | -2.46 | -0.77 |
| P02760 | Protein AMBP (Protein HC) [Cleaved into: Alpha-1-microglobulin (EC 1.6.2.-) (Alpha-1 microglycoprotein) (Complex-forming glycoprotein heterogeneous in charge); Inter-alpha-trypsin inhibitor light chain (ITI-LC) (Bikunin) (EDC1) (HI-30) (Uronic-acid-rich protein); Trypstatin] | AMBP HCP ITIL | unspecified | 8.48E-10 | -5.80 | -2.58 | -5.73 | -2.26 | -0.54 |
| P05543 | Thyroxine-binding globulin (Serpin A7) (T4-binding globulin) | SERPINA7 TBG | non-immune | 1.02E-09 | -5.63 | -3.04 | -5.44 | -2.65 | -0.63 |
| O95445 | Apolipoprotein M (Apo-M) (ApoM) (Protein G3a) | APOM G3A NG20 HSPC336 | non-immune | 1.04E-09 | -5.82 | -2.84 | -5.91 | -2.28 | 0.53 |
| Q5CZ94 | Uncharacterized protein DKFZp781M0386 | DKFZp781M0386 | non-immune | 1.18E-09 | -5.41 | -3.13 | -5.42 | -3.09 | -1.60 |
| Q5FWF9 | IGL@ protein | IGL@ | non-immune | 1.25E-09 | -5.89 | -2.63 | -5.23 | -3.43 | -0.65 |
| A0A087X0Q4 | NA | NA | NA | 1.31E-09 | -4.70 | -3.99 | -5.25 | -3.31 | 0.98 |
| P61952 | Guanine nucleotide-binding protein G(I)/G(S)/G(O) subunit gamma-11 | GNG11 GNGT11 | non-immune | 1.39E-09 | -5.47 | -2.05 | -5.21 | -2.17 | -1.13 |
| A0A0X9V9B3 | MS-F1 light chain variable region |  | unspecified | 2.31E-09 | -4.97 | -3.24 | -5.19 | -2.90 | -0.77 |
| P08697 | Alpha-2-antiplasmin (Alpha-2-AP) (Alpha-2-plasmin inhibitor) (Alpha-2-PI) (Serpin F2) | SERPINF2 AAP PLI | non-immune | 2.96E-09 | -5.72 | -2.39 | -5.01 | -3.09 | -2.23 |
| P0DOX5 | Immunoglobulin gamma-1 heavy chain (Immunoglobulin gamma-1 heavy chain NIE) |  | adaptive | 3.47E-09 | -5.35 | -2.31 | -5.26 | -2.83 | -0.06 |
| P30041 | Peroxiredoxin-6 (EC 1.11.1.27) (1-Cys peroxiredoxin) (1-Cys PRX) (24 kDa protein) (Acidic calcium-independent phospholipase A2) (aiPLA2) (EC 3.1.1.4) (Antioxidant protein 2) (Glutathione-dependent peroxiredoxin) (Liver 2D page spot 40) (Lysophosphatidylcholine acyltransferase 5) (LPC acyltransferase 5) (LPCAT-5) (Lyso-PC acyltransferase 5) (EC 2.3.1.23) (Non-selenium glutathione peroxidase) (NSGPx) (Red blood cells page spot 12) | PRDX6 AOP2 KIAA0106 | non-immune | 3.60E-09 | -5.87 | -1.69 | -5.40 | -1.91 | -1.96 |
| A0A075B6S9 | Probable non-functional immunoglobulinn kappa variable 1-37 | IGKV1-37 | adaptive | 4.29E-09 | -5.12 | -3.06 | -5.25 | -2.57 | -0.27 |
| P80511 | Protein S100-A12 (CGRP) (Calcium-binding protein in amniotic fluid 1) (CAAF1) (Calgranulin-C) (CAGC) (Extracellular newly identified RAGE-binding protein) (EN-RAGE) (Migration inhibitory factor-related protein 6) (MRP-6) (p6) (Neutrophil S100 protein) (S100 calcium-binding protein A12) [Cleaved into: Calcitermin] | S100A12 | innate | 4.56E-09 | 5.23 | 2.41 | 5.26 | 2.10 | 1.06 |
| Q0KKI6 | Immunoblobulin light chain |  | unspecified | 5.25E-09 | -5.57 | -2.43 | -5.87 | -1.88 | -0.01 |
| A0A2U8J954 | Ig heavy chain variable region | IgH | adaptive | 7.52E-09 | -4.92 | -3.24 | -4.97 | -3.21 | 0.43 |
| A0N5G5 | Rheumatoid factor D5 light chain | V-kappa-3 | unspecified | 1.06E-08 | -4.43 | -3.52 | -4.75 | -2.86 | -0.51 |
| P07339 | Cathepsin D (EC 3.4.23.5) [Cleaved into: Cathepsin D light chain; Cathepsin D heavy chain] | CTSD CPSD | unspecified | 1.29E-08 | -5.62 | -2.45 | -5.36 | -2.46 | -1.37 |
| A0A1U9WZ84 | Insulin-like growth factor I |  | non-immune | 1.40E-08 | -5.42 | -2.19 | -5.18 | -2.50 | -1.90 |
| P61224 | Ras-related protein Rap-1b (EC 3.6.5.2) (GTP-binding protein smg p21B) | RAP1B OK/SW-cl.11 | non-immune | 1.45E-08 | -5.22 | -2.42 | -4.93 | -2.50 | -1.27 |
| P00918 | Carbonic anhydrase 2 (EC 4.2.1.1) (Carbonate dehydratase II) (Carbonic anhydrase C) (CAC) (Carbonic anhydrase II) (CA-II) (Cyanamide hydratase CA2) (EC 4.2.1.69) | CA2 | non-immune | 1.52E-08 | -5.34 | -2.82 | -4.97 | -3.11 | -2.00 |
| P10909 | Clusterin (Aging-associated gene 4 protein) (Apolipoprotein J) (Apo-J) (Complement cytolysis inhibitor) (CLI) (Complement-associated protein SP-40,40) (Ku70-binding protein 1) (NA1/NA2) (Sulfated glycoprotein 2) (SGP-2) (Testosterone-repressed prostate message 2) (TRPM-2) [Cleaved into: Clusterin beta chain (ApoJalpha) (Complement cytolysis inhibitor a chain) (SP-40,40 beta-chain); Clusterin alpha chain (ApoJbeta) (Complement cytolysis inhibitor b chain) (SP-40,40 alpha-chain)] | CLU APOJ CLI KUB1 AAG4 | both | 1.65E-08 | -4.93 | -3.06 | -4.60 | -3.32 | -1.31 |
| A0A5C2FT48 | IGL c108_light_IGLV2-11_IGLJ1 |  | unspecified | 1.65E-08 | -5.05 | -2.79 | -5.05 | -2.40 | -1.59 |
| P49720 | Proteasome subunit beta type-3 (Proteasome chain 13) (Proteasome component C10-II) (Proteasome subunit beta-3) (beta-3) (Proteasome theta chain) | PSMB3 | non-immune | 1.89E-08 | -5.67 | -1.56 | -5.07 | -2.13 | -2.32 |
| O19504 | HLA-DRB1*0405V2 specificity |  | unspecified | 2.25E-08 | 5.18 | 2.34 | 5.12 | 2.62 | -0.35 |
| P63104 | 14-3-3 protein zeta/delta (Protein kinase C inhibitor protein 1) (KCIP-1) | YWHAZ | innate | 2.54E-08 | -5.25 | -1.79 | -4.89 | -1.91 | -1.66 |
| Q562R1 | Beta-actin-like protein 2 (Kappa-actin) | ACTBL2 | non-immune | 2.58E-08 | -5.87 | -1.98 | -5.42 | -1.99 | -1.43 |
| Q9Y274 | Type 2 lactosamine alpha-2,3-sialyltransferase (EC 2.4.99.-) (CMP-NeuAc:beta-galactoside alpha-2,3-sialyltransferase VI) (ST3Gal VI) (ST3GalVI) (Sialyltransferase 10) | ST3GAL6 SIAT10 | non-immune | 3.36E-08 | -4.88 | -2.83 | -4.98 | -2.52 | -1.06 |
| P27169 | Serum paraoxonase/arylesterase 1 (PON 1) (EC 3.1.1.2) (EC 3.1.1.81) (EC 3.1.8.1) (Aromatic esterase 1) (A-esterase 1) (K-45) (Serum aryldialkylphosphatase 1) | PON1 PON | non-immune | 3.72E-08 | -5.60 | -1.58 | -4.95 | -1.88 | -0.53 |
| A0A5C2GMK3 | IG c586_heavy_IGHV3-74_IGHD3-10_IGHJ6 |  | adaptive | 3.74E-08 | -4.58 | -3.18 | -4.57 | -2.90 | -0.29 |
| P36955 | Pigment epithelium-derived factor (PEDF) (Cell proliferation-inducing gene 35 protein) (EPC-1) (Serpin F1) | SERPINF1 PEDF PIG35 | non-immune | 4.10E-08 | -5.04 | -2.66 | -4.27 | -3.53 | -0.64 |
| A0A2U8J8R6 | Ig heavy chain variable region | IgH | adaptive | 4.29E-08 | -5.12 | -1.94 | -5.45 | -1.08 | -1.86 |
| P08637 | Low affinity immunoglobulin gamma Fc region receptor III-A (IgG Fc receptor III-A) (CD16-II) (CD16a antigen) (Fc-gamma RIII-alpha) (Fc-gamma RIII) (Fc-gamma RIIIa) (FcRIII) (FcRIIIa) (FcgammaRIIIA) (FcR-10) (IgG Fc receptor III-2) (CD antigen CD16a) | FCGR3A CD16A FCG3 FCGR3 IGFR3 | both | 5.13E-08 | -4.57 | -2.45 | -4.73 | -2.00 | 0.12 |
| S6BGD6 | IgG L chain |  | non-immune | 5.16E-08 | -4.42 | -3.21 | -4.19 | -3.64 | -0.74 |
| P62826 | GTP-binding nuclear protein Ran (EC 3.6.5.-) (Androgen receptor-associated protein 24) (GTPase Ran) (Ras-like protein TC4) (Ras-related nuclear protein) | RAN ARA24 OK/SW-cl.81 | non-immune | 6.43E-08 | -6.62 | -1.24 | -5.83 | -1.78 | -2.83 |
| Q14767 | Latent-transforming growth factor beta-binding protein 2 (LTBP-2) | LTBP2 C14orf141 LTBP3 | non-immune | 9.83E-08 | 5.86 | 1.31 | 5.55 | 1.43 | 0.80 |
| O60888 | Protein CutA (Acetylcholinesterase-associated protein) (Brain acetylcholinesterase putative membrane anchor) | CUTA ACHAP C6orf82 | non-immune | 1.13E-07 | -4.66 | -1.87 | -4.13 | -2.35 | -1.81 |
| Q96RP9 | Elongation factor G, mitochondrial (EF-Gmt) (Elongation factor G 1, mitochondrial) (mEF-G 1) (Elongation factor G1) (hEFG1) | GFM1 EFG EFG1 GFM | non-immune | 1.18E-07 | -4.63 | -2.75 | -4.27 | -3.04 | -0.09 |
| P61970 | Nuclear transport factor 2 (NTF-2) (Placental protein 15) (PP15) | NUTF2 NTF2 | non-immune | 1.48E-07 | -4.62 | -2.26 | -4.57 | -2.08 | -1.79 |
| P04632 | Calpain small subunit 1 (CSS1) (Calcium-activated neutral proteinase small subunit) (CANP small subunit) (Calcium-dependent protease small subunit) (CDPS) (Calcium-dependent protease small subunit 1) (Calpain regulatory subunit) | CAPNS1 CAPN4 CAPNS | non-immune | 1.54E-07 | -5.47 | -1.13 | -4.93 | -1.57 | -1.49 |
| Q9Y509 | VH3 protein | VH3 | adaptive | 1.72E-07 | -4.90 | -2.49 | -4.17 | -3.04 | 1.40 |
| Q7Z351 | Uncharacterized protein DKFZp686N02209 | DKFZp686N02209 | adaptive | 1.92E-07 | -5.19 | -1.88 | -5.54 | -1.09 | -1.69 |
| Q13103 | Secreted phosphoprotein 24 (Spp-24) (Secreted phosphoprotein 2) | SPP2 SPP24 | non-immune | 1.96E-07 | -4.78 | -2.75 | -4.96 | -2.05 | -1.89 |
| B4DUI8 | Actin, aortic smooth muscle (Alpha-actin-2) |  | non-immune | 2.13E-07 | -5.01 | -1.75 | -4.72 | -1.77 | -1.54 |
| A0A087WWU8 | Tropomyosin 3 | TPM3 | non-immune | 2.18E-07 | -4.34 | -1.24 | -4.18 | -1.16 | -0.07 |
| P01137 | Transforming growth factor beta-1 proprotein [Cleaved into: Latency-associated peptide (LAP); Transforming growth factor beta-1 (TGF-beta-1)] | TGFB1 TGFB | both | 2.20E-07 | -4.36 | -2.48 | -4.35 | -2.15 | -1.19 |
| P10720 | Platelet factor 4 variant (C-X-C motif chemokine 4 variant) (CXCL4L1) (PF4alt) (PF4var1) [Cleaved into: Platelet factor 4 variant(4-74); Platelet factor 4 variant(5-74); Platelet factor 4 variant(6-74)] | PF4V1 CXCL4V1 SCYB4V1 | unspecified | 2.52E-07 | -4.10 | -3.29 | -4.17 | -2.77 | -1.47 |
| A0A024R962 | NA | NA | NA | 2.70E-07 | 4.73 | 1.80 | 4.93 | 1.29 | -0.62 |
| P40763 | Signal transducer and activator of transcription 3 (Acute-phase response factor) | STAT3 APRF | adaptive | 2.79E-07 | -3.38 | -5.15 | -4.19 | -3.93 | -0.47 |
| P60709 | Actin, cytoplasmic 1 (EC 3.6.4.-) (Beta-actin) [Cleaved into: Actin, cytoplasmic 1, N-terminally processed] | ACTB | unspecified | 2.82E-07 | -5.11 | -1.52 | -4.66 | -1.77 | -1.59 |
| P78417 | Glutathione S-transferase omega-1 (GSTO-1) (EC 2.5.1.18) (Glutathione S-transferase omega 1-1) (GSTO 1-1) (Glutathione-dependent dehydroascorbate reductase) (EC 1.8.5.1) (Monomethylarsonic acid reductase) (MMA(V) reductase) (EC 1.20.4.2) (S-(Phenacyl)glutathione reductase) (SPG-R) | GSTO1 GSTTLP28 | non-immune | 2.85E-07 | -5.25 | -1.63 | -4.49 | -2.45 | -1.47 |
| A0A5C2FUA9 | IGL c571_light_IGKV3-15_IGKJ2 |  | unspecified | 3.01E-07 | -4.97 | -1.97 | -4.80 | -1.94 | -0.08 |
| P15531 | Nucleoside diphosphate kinase A (NDK A) (NDP kinase A) (EC 2.7.4.6) (Granzyme A-activated DNase) (GAAD) (Metastasis inhibition factor nm23) (NM23-H1) (Tumor metastatic process-associated protein) | NME1 NDPKA NM23 | non-immune | 3.26E-07 | -4.54 | -1.69 | -4.00 | -2.21 | -0.89 |
| P27348 | 14-3-3 protein theta (14-3-3 protein T-cell) (14-3-3 protein tau) (Protein HS1) | YWHAQ | non-immune | 3.43E-07 | -4.56 | -1.85 | -4.14 | -2.43 | -0.18 |
| A0A0X9T7V9 | GCT-A4 light chain variable region |  | non-immune | 3.44E-07 | -4.71 | -2.50 | -3.77 | -3.67 | -0.22 |
| O75396 | Vesicle-trafficking protein SEC22b (ER-Golgi SNARE of 24 kDa) (ERS-24) (ERS24) (SEC22 vesicle-trafficking protein homolog B) (SEC22 vesicle-trafficking protein-like 1) | SEC22B SEC22L1 | non-immune | 3.44E-07 | -4.14 | -4.43 | -4.55 | -3.29 | -3.84 |
| A0A5C2GM39 | IG c248_heavy_IGHV4-34_IGHD2-8_IGHJ6 |  | adaptive | 3.50E-07 | -4.53 | -2.65 | -4.43 | -2.63 | -1.20 |
| P15153 | Ras-related C3 botulinum toxin substrate 2 (GX) (Small G protein) (p21-Rac2) | RAC2 | unspecified | 4.19E-07 | -4.79 | -1.69 | -4.46 | -1.75 | -1.75 |
| P28161 | Glutathione S-transferase Mu 2 (EC 2.5.1.18) (GST class-mu 2) (GSTM2-2) | GSTM2 GST4 | non-immune | 4.30E-07 | -3.03 | -1.87 | -3.23 | -1.53 | -0.83 |
| A0A5C2FV25 | IGL c6_light_IGLV1-44_IGLJ2 |  | unspecified | 4.55E-07 | -4.89 | -1.84 | -4.21 | -2.75 | -0.90 |
| A0A1C9J6R3 | B cell receptor heavy chain variable region |  | adaptive | 6.33E-07 | -4.49 | -2.43 | -4.19 | -2.70 | -0.50 |
| A0A5C2GVK8 | IG c1511_heavy_IGHV4-34_IGHD3-22_IGHJ5 |  | adaptive | 6.37E-07 | -4.68 | -2.05 | -4.51 | -1.73 | -0.26 |
| A0A5C2FZB7 | IGL c2034_light_IGLV2-8_IGLJ2 |  | unspecified | 6.37E-07 | -4.02 | -2.68 | -4.35 | -2.35 | -0.46 |
| Q8N355 | IGL@ protein | IGL@ | non-immune | 6.45E-07 | -5.06 | -2.05 | -4.41 | -2.15 | -1.93 |
| P07195 | L-lactate dehydrogenase B chain (LDH-B) (EC 1.1.1.27) (LDH heart subunit) (LDH-H) (Renal carcinoma antigen NY-REN-46) | LDHB | non-immune | 6.90E-07 | -5.31 | -1.40 | -4.68 | -1.96 | -1.44 |
| A2KBC3 | Anti-(ED-B) scFV |  | adaptive | 7.11E-07 | -3.43 | -4.00 | -3.40 | -4.58 | -0.50 |
| P24593 | Insulin-like growth factor-binding protein 5 (IBP-5) (IGF-binding protein 5) (IGFBP-5) | IGFBP5 IBP5 | non-immune | 7.36E-07 | 3.43 | 3.78 | 4.63 | 2.00 | -1.51 |
| P29622 | Kallistatin (Kallikrein inhibitor) (Peptidase inhibitor 4) (PI-4) (Serpin A4) | SERPINA4 KST PI4 | non-immune | 9.65E-07 | -4.06 | -3.27 | -4.33 | -2.39 | 0.59 |
| A0A5C2GYF2 | IG c668_light_IGLV3-16_IGLJ3 |  | non-immune | 9.82E-07 | -5.21 | -1.86 | -4.51 | -2.09 | -1.12 |
| Q9HCJ5 | Zinc finger SWIM domain-containing protein 6 | ZSWIM6 KIAA1577 | non-immune | 9.85E-07 | 4.16 | 2.20 | 4.60 | 1.82 | -1.87 |
| B3KWB5 | cDNA FLJ42722 fis, clone BRAMY4000277, highly similar to Alpha-1B-glycoprotein |  | non-immune | 9.91E-07 | -3.97 | -3.08 | -3.72 | -2.92 | -1.33 |
| O75460 | Serine/threonine-protein kinase/endoribonuclease IRE1 (Endoplasmic reticulum-to-nucleus signaling 1) (Inositol-requiring protein 1) (hIRE1p) (Ire1-alpha) (IRE1a) [Includes: Serine/threonine-protein kinase (EC 2.7.11.1); Endoribonuclease (EC 3.1.26.-)] | ERN1 IRE1 | non-immune | 1.03E-06 | -4.15 | -2.60 | -4.50 | -1.75 | -0.32 |
| Q7Z7A1 | Centriolin (Centrosomal protein 1) (Centrosomal protein of 110 kDa) (Cep110) | CNTRL CEP1 CEP110 | non-immune | 1.04E-06 | -4.85 | -1.52 | -4.67 | -1.59 | -0.68 |
| Q32Q12 | Nucleoside diphosphate kinase (EC 2.7.4.6) | NME1-NME2 hCG_2001850 | non-immune | 1.05E-06 | -4.63 | -1.24 | -4.36 | -1.24 | -1.65 |
| A2JA16 | Anti-mucin1 light chain variable region |  | unspecified | 1.10E-06 | -3.68 | -3.13 | -3.32 | -3.72 | -0.16 |
| P07737 | Profilin-1 (Epididymis tissue protein Li 184a) (Profilin I) | PFN1 | non-immune | 1.13E-06 | -4.75 | -1.78 | -4.37 | -1.96 | -1.15 |
| P19827 | Inter-alpha-trypsin inhibitor heavy chain H1 (ITI heavy chain H1) (ITI-HC1) (Inter-alpha-inhibitor heavy chain 1) (Inter-alpha-trypsin inhibitor complex component III) (Serum-derived hyaluronan-associated protein) (SHAP) | ITIH1 IGHEP1 | non-immune | 1.14E-06 | -3.62 | -3.63 | -4.21 | -2.37 | -1.60 |
| P63218 | Guanine nucleotide-binding protein G(I)/G(S)/G(O) subunit gamma-5 | GNG5 GNGT5 | non-immune | 1.25E-06 | -4.21 | -1.89 | -4.17 | -1.58 | -2.38 |
| P13501 | C-C motif chemokine 5 (EoCP) (Eosinophil chemotactic cytokine) (SIS-delta) (Small-inducible cytokine A5) (T cell-specific protein P228) (TCP228) (T-cell-specific protein RANTES) [Cleaved into: RANTES(3-68); RANTES(4-68)] | CCL5 D17S136E SCYA5 | innate | 1.43E-06 | -4.22 | -2.21 | -3.81 | -2.85 | -3.31 |
| P20774 | Mimecan (Osteoglycin) (Osteoinductive factor) (OIF) | OGN OIF SLRR3A | non-immune | 1.47E-06 | 4.57 | 3.13 | 5.20 | 1.78 | 0.95 |
| P62834 | Ras-related protein Rap-1A (EC 3.6.5.2) (C21KG) (G-22K) (GTP-binding protein smg p21A) (Ras-related protein Krev-1) | RAP1A KREV1 | non-immune | 1.48E-06 | -4.46 | -2.76 | -4.61 | -2.32 | -2.32 |
| A0A2U8J933 | Ig heavy chain variable region | IgH | adaptive | 1.48E-06 | -4.24 | -2.18 | -3.88 | -2.87 | 0.41 |
| P62979 | Ubiquitin-ribosomal protein eS31 fusion protein (Ubiquitin carboxyl extension protein 80) [Cleaved into: Ubiquitin; Small ribosomal subunit protein eS31 (40S ribosomal protein S27a)] | RPS27A UBA80 UBCEP1 | non-immune | 1.52E-06 | -4.54 | -1.87 | -4.01 | -2.32 | -1.60 |
| O75368 | Adapter SH3BGRL (SH3 domain-binding glutamic acid-rich-like protein 1) | SH3BGRL | non-immune | 1.54E-06 | -3.63 | -1.14 | -3.52 | -1.10 | -1.95 |
| A0A384NL10 | Epididymis secretory sperm binding protein |  | non-immune | 1.66E-06 | 3.86 | 2.57 | 4.02 | 1.72 | -0.28 |
| P84095 | Rho-related GTP-binding protein RhoG | RHOG ARHG | non-immune | 1.97E-06 | -3.35 | -2.64 | -3.61 | -1.86 | -1.73 |
| P0DML2 | Chorionic somatomammotropin hormone 1 (Choriomammotropin) (Lactogen) (Placental lactogen) (PL) | CSH1 | non-immune | 2.08E-06 | 2.57 | 1.79 | 3.11 | 1.04 | -2.10 |
| A0A1W6IYJ9 | N90-VRC38.01 light chain variable region |  | unspecified | 2.19E-06 | -3.25 | -3.91 | -4.06 | -2.38 | -1.18 |
| Q6V1P9 | Protocadherin-23 (Cadherin-27) (Cadherin-like protein CDHJ) (Cadherin-like protein VR8) (Protein dachsous homolog 2) (Protocadherin PCDHJ) | DCHS2 CDH27 CDHJ PCDH23 PCDHJ | non-immune | 2.21E-06 | -3.75 | -2.47 | -4.63 | -0.66 | -1.20 |
| A0A2U8J978 | Ig heavy chain variable region | IgH | adaptive | 2.26E-06 | -4.06 | -2.49 | -4.08 | -2.17 | -0.44 |
| B2RNT9 | UHRF1BP1 protein | UHRF1BP1 | non-immune | 2.28E-06 | -3.94 | -2.34 | -3.79 | -2.55 | -1.57 |
| D9IWP9 | Beta-2-glycoprotein 1 (Apolipoprotein H) (Beta-2-glycoprotein I) |  | non-immune | 2.33E-06 | -3.81 | -2.92 | -3.78 | -2.61 | -0.20 |
| A0A3B3ISA6 | Complement C4B (Chido blood group) | C4B | both | 2.48E-06 | -3.79 | -3.00 | -3.78 | -2.54 | 1.01 |
| P68104 | Elongation factor 1-alpha 1 (EF-1-alpha-1) (EC 3.6.5.-) (Elongation factor Tu) (EF-Tu) (Eukaryotic elongation factor 1 A-1) (eEF1A-1) (Leukocyte receptor cluster member 7) | EEF1A1 EEF1A EF1A LENG7 | non-immune | 2.62E-06 | -4.17 | -2.92 | -4.12 | -2.63 | -1.29 |
| A0A5C2GWI7 | IG c1293_light_IGKV4-1_IGKJ2 |  | unspecified | 2.92E-06 | -3.33 | -3.95 | -3.35 | -4.36 | -0.85 |
| Q15404 | Ras suppressor protein 1 (RSP-1) (Rsu-1) | RSU1 RSP1 | non-immune | 2.95E-06 | -3.44 | -2.58 | -3.61 | -2.74 | -0.96 |
| Q96K68 | cDNA FLJ14473 fis, clone MAMMA1001080, highly similar to Homo sapiens SNC73 protein (SNC73) mRNA |  | non-immune | 2.96E-06 | -3.79 | -2.88 | -3.83 | -2.68 | -0.14 |
| Q6DHW4 | Ig-like domain-containing protein |  | non-immune | 3.06E-06 | -3.59 | -2.92 | -3.81 | -2.65 | -0.65 |
| A0A2Y9CYF2 | Ig heavy chain variable region | IgH | adaptive | 3.08E-06 | -4.26 | -2.33 | -4.20 | -2.14 | -0.82 |
| P61960 | Ubiquitin-fold modifier 1 | UFM1 C13orf20 BM-002 | non-immune | 3.19E-06 | -4.22 | -3.50 | -4.73 | -2.21 | -0.38 |
| C9JEE0 | Immunoglobulin lambda like polypeptide 1 | IGLL1 | non-immune | 3.38E-06 | -4.51 | -2.19 | -4.46 | -1.68 | -1.11 |
| Q86TY3 | Armadillo-like helical domain-containing protein 4 (Upstream of mTORC2 protein) | ARMH4 C14orf37 | non-immune | 3.94E-06 | 3.91 | 2.34 | 4.10 | 1.96 | -0.79 |
| Q99969 | Retinoic acid receptor responder protein 2 (Chemerin) (RAR-responsive protein TIG2) (Tazarotene-induced gene 2 protein) | RARRES2 TIG2 | innate | 3.96E-06 | -4.71 | -1.54 | -4.58 | -1.31 | 0.07 |
| A2IPI2 | HRV Fab N27-VL |  | non-immune | 4.01E-06 | -4.88 | -1.32 | -4.87 | -1.10 | 1.00 |
| P01033 | Metalloproteinase inhibitor 1 (Erythroid-potentiating activity) (EPA) (Fibroblast collagenase inhibitor) (Collagenase inhibitor) (Tissue inhibitor of metalloproteinases 1) (TIMP-1) | TIMP1 CLGI TIMP | non-immune | 4.04E-06 | -4.66 | -1.95 | -4.45 | -1.89 | 0.50 |
| P55072 | Transitional endoplasmic reticulum ATPase (TER ATPase) (EC 3.6.4.6) (15S Mg(2+)-ATPase p97 subunit) (Valosin-containing protein) (VCP) | VCP HEL-220 HEL-S-70 | non-immune | 4.12E-06 | -3.58 | -3.16 | -3.79 | -2.69 | -1.89 |
| P04406 | Glyceraldehyde-3-phosphate dehydrogenase (GAPDH) (EC 1.2.1.12) (Peptidyl-cysteine S-nitrosylase GAPDH) (EC 2.6.99.-) | GAPDH GAPD CDABP0047 OK/SW-cl.12 | innate | 4.38E-06 | -4.47 | -1.75 | -4.12 | -1.87 | -1.85 |
| A0A2U8J915 | Ig heavy chain variable region | IgH | adaptive | 4.41E-06 | -3.74 | -2.38 | -4.07 | -1.55 | 1.79 |
| Q56G89 | Albumin |  | non-immune | 4.57E-06 | -4.12 | -1.65 | -3.82 | -2.52 | 0.45 |
| B4E1D8 | cDNA FLJ51597, highly similar to C4b-binding protein alpha chain |  | adaptive | 4.68E-06 | -4.28 | -2.47 | -4.09 | -2.01 | 0.71 |
| P07741 | Adenine phosphoribosyltransferase (APRT) (EC 2.4.2.7) | APRT | non-immune | 4.89E-06 | -4.37 | -3.37 | -4.51 | -3.13 | -1.39 |
| A0A5C2FVS3 | IGL c912_light_IGKV1D-13_IGKJ4 |  | unspecified | 4.94E-06 | -4.92 | -0.85 | -4.89 | -0.49 | -0.63 |
| P63000 | Ras-related C3 botulinum toxin substrate 1 (EC 3.6.5.2) (Cell migration-inducing gene 5 protein) (Ras-like protein TC25) (p21-Rac1) | RAC1 TC25 MIG5 | unspecified | 5.11E-06 | -4.65 | -3.79 | -4.84 | -3.26 | -2.98 |
| V9HW34 | Epididymis luminal protein 213 | HEL-213 | unspecified | 5.34E-06 | -3.93 | -2.42 | -3.67 | -2.59 | -0.02 |
| B4E1Z4 | Complement C2 (EC 3.4.21.43) (C3/C5 convertase) |  | both | 5.36E-06 | -3.60 | -3.06 | -3.50 | -2.62 | -0.28 |
| P60985 | Keratinocyte differentiation-associated protein | KRTDAP KDAP UNQ467/PRO826 | non-immune | 5.53E-06 | 4.48 | 1.33 | 4.25 | 1.53 | 0.82 |
| P0DP23 | Calmodulin-1 | CALM1 CALM CAM CAM1 | innate | 5.58E-06 | -3.62 | -2.35 | -3.37 | -2.41 | -1.98 |
| Q96JD1 | Amyloid lambda 6 light chain variable region PIP |  | unspecified | 5.77E-06 | -3.47 | -2.87 | -3.55 | -2.90 | -0.51 |
| A2J1N7 | Rheumatoid factor RF-ET10 |  | adaptive | 5.97E-06 | -3.76 | -2.88 | -3.77 | -2.36 | -0.47 |
| Q6ZRK6 | Coiled-coil domain-containing protein 73 (Sarcoma antigen NY-SAR-79) | CCDC73 | non-immune | 6.09E-06 | -3.61 | -2.21 | -3.27 | -2.60 | 0.04 |
| P02750 | Leucine-rich alpha-2-glycoprotein (LRG) | LRG1 LRG | non-immune | 6.39E-06 | -3.68 | -2.78 | -3.48 | -2.92 | 0.23 |
| P22792 | Carboxypeptidase N subunit 2 (Carboxypeptidase N 83 kDa chain) (Carboxypeptidase N large subunit) (Carboxypeptidase N polypeptide 2) (Carboxypeptidase N regulatory subunit) | CPN2 ACBP | non-immune | 6.47E-06 | -4.19 | -2.10 | -4.33 | -1.74 | -0.76 |
| P43251 | Biotinidase (Biotinase) (EC 3.5.1.12) | BTD | non-immune | 6.70E-06 | -3.83 | -2.16 | -3.83 | -1.73 | -0.05 |
| A0A2U8J8T6 | Ig heavy chain variable region | IgH | adaptive | 6.81E-06 | -4.35 | -1.62 | -4.07 | -1.99 | -0.40 |
| P62258 | 14-3-3 protein epsilon (14-3-3E) | YWHAE | innate | 6.83E-06 | -4.94 | -0.82 | -4.38 | -1.27 | -1.91 |
| P21333 | Filamin-A (FLN-A) (Actin-binding protein 280) (ABP-280) (Alpha-filamin) (Endothelial actin-binding protein) (Filamin-1) (Non-muscle filamin) | FLNA FLN FLN1 | non-immune | 6.91E-06 | -5.14 | -2.93 | -5.05 | -2.47 | -2.70 |
| P07108 | Acyl-CoA-binding protein (ACBP) (Diazepam-binding inhibitor) (DBI) (Endozepine) (EP) | DBI | non-immune | 7.29E-06 | -3.76 | -2.06 | -3.58 | -2.35 | -1.81 |
| A0A1B0GTC6 | Uncharacterized protein C3orf85 | C3orf85 | non-immune | 7.31E-06 | 4.48 | 1.07 | 4.75 | 0.49 | -0.96 |
| P28300 | Protein-lysine 6-oxidase (EC 1.4.3.13) (Lysyl oxidase) [Cleaved into: Protein-lysine 6-oxidase, long form; Protein-lysine 6-oxidase, short form] | LOX | unspecified | 7.34E-06 | 4.23 | 2.25 | 4.43 | 2.04 | 1.20 |
| A0A2U8J8X8 | Ig heavy chain variable region | IgH | adaptive | 7.54E-06 | -4.43 | -1.55 | -3.92 | -1.98 | -0.40 |
| Q96N23 | Cilia- and flagella-associated protein 54 | CFAP54 C12orf55 C12orf63 | non-immune | 7.80E-06 | -3.48 | -2.13 | -3.45 | -2.17 | -2.05 |
| Q9NP80 | Calcium-independent phospholipase A2-gamma (EC 3.1.1.-) (EC 3.1.1.5) (Intracellular membrane-associated calcium-independent phospholipase A2 gamma) (iPLA2-gamma) (PNPLA-gamma) (Patatin-like phospholipase domain-containing protein 8) (iPLA2-2) | PNPLA8 IPLA22 IPLA2G BM-043 | non-immune | 8.73E-06 | -4.69 | -1.06 | -4.20 | -1.04 | -1.35 |
| P37802 | Transgelin-2 (Epididymis tissue protein Li 7e) (SM22-alpha homolog) | TAGLN2 KIAA0120 CDABP0035 | non-immune | 9.02E-06 | -4.22 | -1.45 | -3.84 | -1.58 | -1.36 |
| M0R2W8 | Peptidoglycan recognition protein 2 | PGLYRP2 | non-immune | 9.49E-06 | -4.73 | -3.12 | -4.67 | -3.17 | -2.21 |
| Q7Z478 | ATP-dependent RNA helicase DHX29 (EC 3.6.4.13) (DEAH box protein 29) (Nucleic acid helicase DDXx) | DHX29 DDX29 | non-immune | 9.85E-06 | -3.57 | -2.85 | -3.55 | -2.67 | -1.32 |
| Q6PIQ7 | IGL@ protein | IGL@ | unspecified | 9.99E-06 | -4.01 | -2.17 | -4.29 | -1.73 | -2.72 |
| P23528 | Cofilin-1 (18 kDa phosphoprotein) (p18) (Cofilin, non-muscle isoform) | CFL1 CFL | non-immune | 1.07E-05 | -4.43 | -1.38 | -4.02 | -1.52 | -2.02 |
| A0A5C2GNV2 | IG c346_light_IGKV3-20_IGKJ2 |  | non-immune | 1.26E-05 | -3.54 | -2.62 | -3.49 | -2.67 | 1.44 |
| A0A120HG39 | MS-A3 heavy chain variable region |  | adaptive | 1.30E-05 | -3.40 | -2.56 | -3.82 | -1.50 | -1.64 |
| Q7Z3Y4 | Ig-like domain-containing protein |  | non-immune | 1.34E-05 | -3.84 | -2.19 | -3.88 | -2.32 | -1.55 |
| Q3ZCW2 | Galectin-related protein (Galectin-like protein) (Lectin galactoside-binding-like protein) | LGALSL GRP HSPC159 | non-immune | 1.37E-05 | -4.71 | -4.14 | -5.10 | -3.60 | -4.06 |
| P0DOX7 | Immunoglobulin kappa light chain (Immunoglobulin kappa light chain EU) |  | adaptive | 1.38E-05 | -4.51 | -1.42 | -4.36 | -1.45 | 0.44 |
| Q6EMK4 | Vasorin (Protein slit-like 2) | VASN SLITL2 UNQ314/PRO357/PRO1282 | non-immune | 1.52E-05 | -3.45 | -2.68 | -3.29 | -2.91 | -0.39 |
| Q9UQF0 | Syncytin-1 (Endogenous retrovirus group W member 1) (Env-W) (Envelope polyprotein gPr73) (Enverin) (HERV-7q Envelope protein) (HERV-W envelope protein) (HERV-W_7q21.2 provirus ancestral Env polyprotein) (Syncytin) [Cleaved into: Surface protein (SU) (gp50); Transmembrane protein (TM) (gp24)] | ERVW-1 ERVWE1 | non-immune | 1.60E-05 | -3.64 | -2.60 | -3.72 | -1.87 | -1.27 |
| Q12907 | Vesicular integral-membrane protein VIP36 (Glycoprotein GP36b) (Lectin mannose-binding 2) (Vesicular integral-membrane protein 36) (VIP36) | LMAN2 C5orf8 | non-immune | 1.65E-05 | -1.95 | -4.66 | -2.40 | -4.37 | -1.05 |
| P99999 | Cytochrome c | CYCS CYC | non-immune | 1.66E-05 | -3.70 | -1.82 | -3.54 | -2.04 | -1.26 |
| O15519 | CASP8 and FADD-like apoptosis regulator (Caspase homolog) (CASH) (Caspase-eight-related protein) (Casper) (Caspase-like apoptosis regulatory protein) (CLARP) (Cellular FLICE-like inhibitory protein) (c-FLIP) (FADD-like antiapoptotic molecule 1) (FLAME-1) (Inhibitor of FLICE) (I-FLICE) (MACH-related inducer of toxicity) (MRIT) (Usurpin) [Cleaved into: CASP8 and FADD-like apoptosis regulator subunit p43; CASP8 and FADD-like apoptosis regulator subunit p12] | CFLAR CASH CASP8AP1 CLARP MRIT | unspecified | 1.70E-05 | -2.62 | -3.32 | -3.23 | -2.04 | -0.59 |
| P62328 | Thymosin beta-4 (T beta-4) (Fx) [Cleaved into: Hemoregulatory peptide AcSDKP (Ac-Ser-Asp-Lys-Pro) (N-acetyl-SDKP) (AcSDKP) (Seraspenide)] | TMSB4X TB4X THYB4 TMSB4 | non-immune | 1.72E-05 | -3.77 | -1.87 | -3.60 | -1.97 | -1.19 |
| P12955 | Xaa-Pro dipeptidase (X-Pro dipeptidase) (EC 3.4.13.9) (Imidodipeptidase) (Peptidase D) (Proline dipeptidase) (Prolidase) | PEPD PRD | non-immune | 1.77E-05 | -4.51 | -1.76 | -4.90 | -0.96 | -0.65 |
| P0DOX2 | Immunoglobulin alpha-2 heavy chain (Immunoglobulin alpha-2 heavy chain BUT) |  | adaptive | 1.88E-05 | -3.10 | -3.37 | -3.03 | -4.00 | -0.98 |
| Q9UL84 | Myosin-reactive immunoglobulin heavy chain variable region |  | adaptive | 1.90E-05 | -4.58 | -0.67 | -4.03 | -1.22 | 1.09 |
| Q4VAM3 | ACTN3 protein | ACTN3 | non-immune | 1.96E-05 | -3.20 | -3.41 | -3.21 | -2.98 | -1.72 |
| Q53YY1 | Angiotensinogen (Serpin A8) | AGT | non-immune | 2.02E-05 | -3.89 | -1.89 | -3.72 | -2.14 | -0.28 |
| P80188 | Neutrophil gelatinase-associated lipocalin (NGAL) (25 kDa alpha-2-microglobulin-related subunit of MMP-9) (Lipocalin-2) (Oncogene 24p3) (Siderocalin) (p25) | LCN2 HNL NGAL | innate | 2.04E-05 | -3.86 | -1.13 | -3.52 | -1.57 | -0.63 |
| A0A125U0V4 | GCT-A2 heavy chain variable region |  | adaptive | 2.12E-05 | -2.98 | -3.13 | -3.65 | -1.93 | 0.25 |
| Q04760 | Lactoylglutathione lyase (EC 4.4.1.5) (Aldoketomutase) (Glyoxalase I) (Glx I) (Ketone-aldehyde mutase) (Methylglyoxalase) (S-D-lactoylglutathione methylglyoxal lyase) | GLO1 | non-immune | 2.16E-05 | -2.14 | -1.03 | -2.28 | -0.58 | -0.72 |
| F8VQD4 | Chromosome 12 open reading frame 75 | C12orf75 | non-immune | 2.17E-05 | -4.57 | -0.51 | -4.25 | -0.62 | -1.46 |
| A0A5C2G2H1 | IGL c3488_light_IGKV1-39_IGKJ2 |  | unspecified | 2.24E-05 | -3.90 | -2.07 | -3.67 | -2.12 | -1.28 |
| A0A2H4G024 | MHC class I antigen | HLA-A | adaptive | 2.25E-05 | -4.57 | -2.28 | -4.54 | -2.05 | 0.36 |
| Q14766 | Latent-transforming growth factor beta-binding protein 1 (LTBP-1) (Transforming growth factor beta-1-binding protein 1) (TGF-beta1-BP-1) | LTBP1 | non-immune | 2.28E-05 | -4.30 | -1.11 | -4.00 | -1.02 | -0.82 |
| P26641 | Elongation factor 1-gamma (EF-1-gamma) (eEF-1B gamma) | EEF1G EF1G PRO1608 | non-immune | 2.53E-05 | -4.14 | -1.30 | -4.63 | -0.19 | -0.81 |
| Q9H4B7 | Tubulin beta-1 chain | TUBB1 | non-immune | 2.56E-05 | -4.02 | -2.20 | -3.83 | -1.98 | -3.25 |
| P03952 | Plasma kallikrein (EC 3.4.21.34) (Fletcher factor) (Kininogenin) (Plasma prekallikrein) (PKK) [Cleaved into: Plasma kallikrein heavy chain; Plasma kallikrein light chain] | KLKB1 KLK3 | non-immune | 2.60E-05 | -4.18 | -1.57 | -4.11 | -1.10 | -1.20 |
| P09603 | Macrophage colony-stimulating factor 1 (CSF-1) (M-CSF) (MCSF) (Lanimostim) (Proteoglycan macrophage colony-stimulating factor) (PG-M-CSF) [Cleaved into: Processed macrophage colony-stimulating factor 1; Macrophage colony-stimulating factor 1 43 kDa subunit] | CSF1 | innate | 2.66E-05 | 4.37 | 0.95 | 3.55 | 2.14 | 0.21 |
| A0A5C2GMX8 | IG c494_heavy_IGHV3-23_IGHD4-17_IGHJ4 |  | adaptive | 2.69E-05 | -4.15 | -1.78 | -3.93 | -1.46 | 0.38 |
| Q01082 | Spectrin beta chain, non-erythrocytic 1 (Beta-II spectrin) (Fodrin beta chain) (Spectrin, non-erythroid beta chain 1) | SPTBN1 SPTB2 | non-immune | 2.92E-05 | -3.00 | -1.48 | -3.21 | -0.78 | -0.43 |
| Q1HP67 | Lipoprotein, Lp(A) | LPA | non-immune | 2.99E-05 | -4.26 | -1.36 | -3.79 | -1.78 | 0.35 |
| M0R0Q9 | Complement C3 | C3 | non-immune | 3.15E-05 | 2.65 | 3.15 | 3.37 | 1.85 | 1.39 |
| A0A5C2GGG9 | IG c256_heavy_IGHV3-33_IGHD3-9_IGHJ6 |  | adaptive | 3.17E-05 | -3.71 | -2.19 | -3.81 | -1.94 | -0.50 |
| P02747 | Complement C1q subcomponent subunit C | C1QC C1QG | both | 3.43E-05 | -3.82 | -1.64 | -3.68 | -1.76 | -0.48 |
| Q8TE63 | Immunglobulin light chain variable region |  | unspecified | 3.57E-05 | -3.48 | -2.16 | -3.53 | -2.22 | -1.32 |
| Q6N097 | Uncharacterized protein DKFZp686H20196 | DKFZp686H20196 | adaptive | 3.65E-05 | 3.48 | 3.30 | 3.85 | 2.51 | 2.53 |
| P12830 | Cadherin-1 (CAM 120/80) (Epithelial cadherin) (E-cadherin) (Uvomorulin) (CD antigen CD324) [Cleaved into: E-Cad/CTF1; E-Cad/CTF2; E-Cad/CTF3] | CDH1 CDHE UVO | non-immune | 3.69E-05 | -4.25 | -1.76 | -3.48 | -2.14 | -0.57 |
| B3KRJ8 | cDNA FLJ34426 fis, clone HHDPC2008474, moderately similar to Insulin-like growth factor-binding protein 3 |  | non-immune | 3.92E-05 | -3.11 | -0.31 | -2.74 | -0.71 | -1.15 |
| Q8N3L3 | Beta-taxilin (Muscle-derived protein 77) (hMDP77) | TXLNB C6orf198 MDP77 | non-immune | 3.95E-05 | 3.58 | 1.41 | 3.85 | 0.84 | -0.38 |
| P37235 | Hippocalcin-like protein 1 (Calcium-binding protein BDR-1) (HLP2) (Visinin-like protein 3) (VILIP-3) | HPCAL1 BDR1 | non-immune | 4.00E-05 | -4.71 | -0.78 | -4.52 | -1.02 | -1.93 |
| P62937 | Peptidyl-prolyl cis-trans isomerase A (PPIase A) (EC 5.2.1.8) (Cyclophilin A) (Cyclosporin A-binding protein) (Rotamase A) [Cleaved into: Peptidyl-prolyl cis-trans isomerase A, N-terminally processed] | PPIA CYPA | unspecified | 4.13E-05 | -4.21 | -1.05 | -3.66 | -1.40 | -1.83 |
| Q9H299 | SH3 domain-binding glutamic acid-rich-like protein 3 (SH3 domain-binding protein 1) (SH3BP-1) (TNF inhibitory protein B1) (TIP-B1) | SH3BGRL3 P1725 | non-immune | 4.37E-05 | -4.01 | -1.32 | -3.53 | -1.63 | -1.89 |
| A0A0X9T7T4 | MS-D3 heavy chain variable region |  | adaptive | 4.40E-05 | -3.72 | -1.79 | -4.07 | -1.14 | -0.44 |
| A0A5C2GU38 | IG c17_heavy_IGHV3-11_IGHD4-17_IGHJ4 |  | adaptive | 4.45E-05 | -3.24 | -2.47 | -3.34 | -2.24 | -0.27 |
| Q9UK54 | Hemoglobin beta subunit variant | HBB | non-immune | 4.62E-05 | -4.23 | -0.95 | -3.61 | -1.56 | -1.81 |
| B6EDE2 | Epididymis luminal protein 180 | HEL180 | adaptive | 4.72E-05 | -3.22 | -2.51 | -3.26 | -2.54 | -0.90 |
| Q99439 | Calponin-2 (Calponin H2, smooth muscle) (Neutral calponin) | CNN2 | unspecified | 4.90E-05 | -3.51 | -2.88 | -3.88 | -1.97 | -1.47 |
| P05121 | Plasminogen activator inhibitor 1 (PAI) (PAI-1) (Endothelial plasminogen activator inhibitor) (Serpin E1) | SERPINE1 PAI1 PLANH1 | unspecified | 5.30E-05 | -3.34 | -3.77 | -3.60 | -3.71 | -1.36 |
| P68366 | Tubulin alpha-4A chain (EC 3.6.5.-) (Alpha-tubulin 1) (Testis-specific alpha-tubulin) (Tubulin H2-alpha) (Tubulin alpha-1 chain) | TUBA4A TUBA1 | non-immune | 5.48E-05 | -4.98 | -2.48 | -4.74 | -2.37 | -2.59 |
| A0A5C2G374 | IGL c3728_light_IGKV4-1_IGKJ1 |  | unspecified | 5.99E-05 | -3.77 | -1.86 | -3.49 | -2.49 | -0.55 |
| Q96QU1 | Protocadherin-15 | PCDH15 USH1F | non-immune | 6.05E-05 | -4.23 | -1.36 | -3.90 | -1.70 | -1.10 |
| Q8NEJ1 | Ig-like domain-containing protein |  | non-immune | 8.14E-05 | -3.44 | -1.74 | -3.37 | -1.85 | 0.90 |
| Q13418 | Integrin-linked protein kinase (EC 2.7.11.1) (59 kDa serine/threonine-protein kinase) (Beta-integrin-linked kinase) (ILK-1) (ILK-2) (p59ILK) | ILK ILK1 ILK2 | non-immune | 0.00012 | -4.20 | -1.46 | -4.30 | -0.86 | -2.80 |
| O95810 | Caveolae-associated protein 2 (Cavin-2) (PS-p68) (Phosphatidylserine-binding protein) (Serum deprivation-response protein) | CAVIN2 SDPR | non-immune | 0.00014 | -3.96 | -1.89 | -3.83 | -1.59 | -1.09 |
| S6BAM6 | IgG H chain |  | adaptive | 0.00017 | -2.62 | -3.50 | -2.93 | -3.13 | -0.05 |
| O14786 | Neuropilin-1 (Vascular endothelial cell growth factor 165 receptor) (CD antigen CD304) | NRP1 NRP VEGF165R | non-immune | 0.0002 | -3.63 | -1.89 | -3.57 | -2.12 | 1.23 |
| Q9UBW5 | Bridging integrator 2 (Breast cancer-associated protein 1) | BIN2 BRAP1 | non-immune | 0.00161 | -4.21 | -1.52 | -4.11 | -1.08 | -1.00 |
| O00602 | Ficolin-1 (Collagen/fibrinogen domain-containing protein 1) (Ficolin-A) (Ficolin-alpha) (M-ficolin) | FCN1 FCNM | innate | 0.05752 | -3.16 | -4.68 | -3.74 | -3.50 | -2.92 |

Supplemental Table S2. Topological metrics of protein-protein correlation networks, stratified by occupation and algorithm.

| Occupation | Tree/algorithm | Protein Type | Nodes | Edges | Average Neighbors | Diameter | Radius | Clustering Coefficient | Density | Heterogeneity | Centralization |
| --- | --- | --- | --- | --- | --- | --- | --- | --- | --- | --- | --- |
| cotton | Prim's MST | All | 224 | 223 | 1.991 | 30 | 15 | 0 | 0.009 | 0.904 | 0.05 |
| silk | Prim's MST | All | 224 | 223 | 1.991 | 25 | 13 | 0 | 0.009 | 0.821 | 0.041 |
| cotton | Prim's MST | Immune | 96 | 95 | 1.979 | 13 | 7 | 0 | 0.021 | 1.151 | 0.161 |
| silk | Prim's MST | Immune | 96 | 95 | 1.979 | 16 | 6 | 0 | 0.021 | 0.819 | 0.108 |
| cotton | TMFG | All | 224 | 666 | 5.946 | 8 | 4 | 0.751 | 0.027 | 0.876 | 0.168 |
| silk | TMFG | All | 224 | 666 | 5.946 | 9 | 5 | 0.747 | 0.027 | 0.827 | 0.113 |
| cotton | TMFG | Immune | 96 | 282 | 5.875 | 7 | 4 | 0.755 | 0.062 | 0.828 | 0.062 |
| silk | TMFG | Immune | 96 | 282 | 5.875 | 7 | 4 | 0.755 | 0.062 | 0.786 | 0.216 |

Definition of abbreviations: MST=Maximum Spinning Tree; TMFG=Triangulated Maximally Filtered Graph

Supplemental Table S3. Top enriched Gene Ontology (GO), KEGG, and Reactome Pathways of differentially expressed proteins between endotoxin-exposed and control groups

| Category | Term ID | Term Description | Proportion  Downregulated  (p-value) | Normalized Enrichment Score (p-value) | observed/background gene | false discovery rate | signal |
| --- | --- | --- | --- | --- | --- | --- | --- |
| GO Process | GO:0052548 | Regulation of endopeptidase activity | 95% (0.00) | -0.67 (0.9) | 20/414 | 1.20E-07 | 1.28 |
| GO Process | GO:0051346 | Negative regulation of hydrolase activity | 88% (0.01) | 1.08 (0.49) | 16/354 | 8.48E-06 | 1.01 |
| GO Process | GO:0006954 | Inflammatory response | 85% (0.01) | -0.44 (0.98) | 20/538 | 2.88E-06 | 1.01 |
| GO Process | GO:0050921 | Positive regulation of chemotaxis | 90% (0.01) | -1.51 (0.09) | 10/147 | 8.30E-05 | 0.95 |
| GO Process | GO:0030162 | Regulation of proteolysis | 95% (0.00) | 0.97 (0.57) | 22/739 | 1.22E-05 | 0.86 |
| GO Process | GO:0042060 | Wound healing | 93% (0.00) | -1.39 (0.18) | 14/336 | 8.30E-05 | 0.85 |
| GO Process | GO:0006911 | Phagocytosis, engulfment | 100% (0.03) | -1.68 (0.02) | 6/51 | 7.20E-04 | 0.82 |
| GO Process | GO:0032103 | Positive regulation of response to external stimulus | 81% (0.02) | -1.35 (0.2) | 16/453 | 8.30E-05 | 0.81 |
| GO Process | GO:0002526 | Acute inflammatory response | 100% (0.02) | 1.13 (0.42) | 7/80 | 6.50E-04 | 0.8 |
| GO Process | GO:0051050 | Positive regulation of transport | 96% (0.00) | -1.64 (0.04) | 24/915 | 2.29E-05 | 0.79 |
| GO Process | GO:0030036 | Actin cytoskeleton organization | 100% (0.00) | -2.05 (0) | 17/547 | 1.50E-04 | 0.74 |
| GO Process | GO:0060627 | Regulation of vesicle-mediated transport | 88% (0.01) | -1.42 (0.16) | 17/551 | 1.60E-04 | 0.74 |
| GO Process | GO:0002460 | Adaptive immune response based on somatic recombination of immune receptors built from immunoglobulin superfamily domains | 89% (0.02) | -0.51 (0.98) | 9/169 | 9.90E-04 | 0.71 |
| GO Process | GO:0030168 | Platelet activation | 100% (0.02) | -1.5 (0.11) | 7/97 | 1.50E-03 | 0.7 |
| GO Process | GO:0032760 | Positive regulation of tumor necrosis factor production | 100% (0.02) | -0.76 (0.84) | 7/101 | 1.80E-03 | 0.68 |
| GO Process | GO:0050764 | Regulation of phagocytosis | 86% (0.05) | -0.81 (0.8) | 7/104 | 2.00E-03 | 0.67 |
| GO Process | GO:0002685 | Regulation of leukocyte migration | 90% (0.01) | -1.72 (0.02) | 10/231 | 1.40E-03 | 0.65 |
| GO Process | GO:0002687 | Positive regulation of leukocyte migration | 88% (0.04) | -1.47 (0.11) | 8/151 | 2.30E-03 | 0.63 |
| GO Process | GO:0043086 | Negative regulation of catalytic activity | 84% (0.02) | 1.04 (0.56) | 19/771 | 5.40E-04 | 0.62 |
| GO Component | GO:0072562 | Blood microparticle | 94% (0.00) | -0.85 (0.74) | 18/118 | 3.54E-16 | 3.18 |
| GO Component | GO:0034774 | Secretory granule lumen | 93% (0.00) | -0.84 (0.76) | 27/321 | 1.23E-18 | 2.82 |
| GO Component | GO:0070062 | Extracellular exosome | 94% (0.00) | -1.94 (0) | 80/2096 | 2.04E-39 | 2.26 |
| GO Component | GO:0031093 | Platelet alpha granule lumen | 100% (0.00) | -0.8 (0.79) | 11/66 | 4.98E-10 | 2.19 |
| GO Component | GO:0062023 | Collagen-containing extracellular matrix | 84% (0.01) | 1.1 (0.46) | 25/407 | 2.93E-14 | 2.1 |
| GO Component | GO:0005615 | Extracellular space | 89% (0.00) | -1.89 (0) | 99/3247 | 2.37E-44 | 1.92 |
| GO Component | GO:0031012 | Extracellular matrix | 85% (0.01) | 1.18 (0.42) | 27/552 | 2.88E-13 | 1.81 |
| GO Component | GO:0030141 | Secretory granule | 88% (0.00) | -1.2 (0.35) | 33/873 | 1.97E-13 | 1.59 |
| GO Component | GO:0031982 | Vesicle | 91% (0.00) | -2.01 (0) | 94/3957 | 1.49E-32 | 1.48 |
| GO Component | GO:0099503 | Secretory vesicle | 88% (0.00) | -1.2 (0.35) | 34/1047 | 4.00E-12 | 1.39 |
| GO Component | GO:0005925 | Focal adhesion | 100% (0.00) | -2.22 (0) | 16/416 | 3.91E-06 | 1.01 |
| GO Component | GO:0097708 | Intracellular vesicle | 88% (0.00) | -1.3 (0.21) | 49/2484 | 2.95E-10 | 0.96 |
| GO Component | GO:0031410 | Cytoplasmic vesicle | 87% (0.00) | -1.33 (0.19) | 48/2482 | 9.98E-10 | 0.92 |
| GO Component | GO:0042470 | Melanosome | 100% (0.02) | -1.57 (0.05) | 7/109 | 1.10E-03 | 0.72 |
| GO Function | GO:0004866 | Endopeptidase inhibitor activity | 93% (0.00) | 0.82 (0.82) | 15/177 | 1.95E-08 | 1.64 |

| GO Function | GO:0061134 | Peptidase regulator activity | 94% (0.00) | 0.91 (0.71) | 16/227 | 1.95E-08 | 1.55 |
| --- | --- | --- | --- | --- | --- | --- | --- |
| GO Function | GO:0004867 | Serine-type endopeptidase inhibitor activity | 100% (0.00) | 1.2 (0.32) | 10/98 | 2.75E-06 | 1.32 |
| GO Function | GO:0098772 | Molecular function regulator activity | 84% (0.00) | -1.25 (0.29) | 38/1960 | 2.75E-06 | 0.76 |
| GO Function | GO:0045296 | Cadherin binding | 100% (0.00) | -1.78 (0.02) | 13/334 | 3.00E-04 | 0.75 |
| GO Function | GO:0030234 | Enzyme regulator activity | 88% (0.00) | -0.59 (0.93) | 26/1239 | 1.70E-04 | 0.64 |
| GO Function | GO:0005102 | Signaling receptor binding | 79% (0.03) | 1.28 (0.32) | 29/1499 | 1.70E-04 | 0.62 |
| GO Function | GO:0019899 | Enzyme binding | 100% (0.00) | -2.03 (0) | 36/2084 | 8.33E-05 | 0.61 |
| GO Function | GO:0003924 | GTPase activity | 100% (0.00) | -2 (0) | 11/317 | 5.50E-03 | 0.52 |
| GO Function | GO:0019901 | Protein kinase binding | 100% (0.00) | -2.11 (0) | 17/702 | 3.00E-03 | 0.52 |
| GO Function | GO:0005515 | Protein binding | 89% (0.00) | -1.94 (0) | 81/7242 | 2.50E-05 | 0.51 |
| GO Function | GO:0005525 | GTP binding | 100% (0.00) | -1.98 (0) | 12/381 | 5.50E-03 | 0.51 |
| KEGG | hsa04610 | Complement and coagulation cascades | 89% (0.04) | 1.53 (0.17) | 9/82 | 3.94E-06 | 1.3 |
| KEGG | hsa04062 | Chemokine signaling pathway | 100% (0.00) | -1.75 (0.02) | 11/186 | 1.81E-05 | 1.04 |
| KEGG | hsa05142 | Chagas disease | 86% (0.05) | -0.92 (0.65) | 7/97 | 4.80E-04 | 0.81 |
| KEGG | hsa05131 | Shigellosis | 90% (0.00) | -1.69 (0.03) | 10/218 | 4.10E-04 | 0.76 |
| KEGG | hsa04015 | Rap1 signaling pathway | 88% (0.02) | -1.89 (0.01) | 9/201 | 7.80E-04 | 0.7 |
| KEGG | hsa04014 | Ras signaling pathway | 88% (0.02) | -1.85 (0.01) | 9/225 | 1.40E-03 | 0.64 |
| KEGG | hsa05167 | Kaposi sarcoma-associated herpesvirus infection | 86% (0.03) | -1.23 (0.35) | 8/187 | 2.20E-03 | 0.61 |
| KEGG | hsa04390 | Hippo signaling pathway | 100% (0.02) | -1.5 (0.11) | 7/154 | 3.60E-03 | 0.58 |
| KEGG | hsa04670 | Leukocyte transendothelial migration | 100% (0.03) | -1.79 (0.01) | 6/111 | 4.20E-03 | 0.58 |
| KEGG | hsa05200 | Pathways in cancer | 100% (0.00) | -1.67 (0.04) | 13/515 | 2.20E-03 | 0.54 |
| KEGG | hsa04210 | Apoptosis | 100% (0.03) | -1.21 (0.35) | 6/131 | 7.90E-03 | 0.51 |
| Reactome | HSA-76002 | Platelet activation, signaling, and aggregation | 100% (0.00) | -2.15 (0) | 31/260 | 5.41E-25 | 3.9 |
| Reactome | HSA-114608 | Platelet degranulation | 100% (0.00) | -1.34 (0.23) | 21/126 | 3.29E-19 | 3.71 |
| Reactome | HSA-109582 | Hemostasis | 100% (0.00) | -2.15 (0) | 36/607 | 4.09E-20 | 2.46 |
| Reactome | HSA-5626467 | RHO GTPases activate IQGAPs | 100% (0.03) | -1.99 (0) | 7/31 | 1.40E-06 | 1.52 |
| Reactome | HSA-168249 | Innate Immune System | 94% (0.00) | -1.47 (0.1) | 35/1041 | 1.66E-12 | 1.44 |
| Reactome | HSA-6798695 | Neutrophil degranulation | 90% (0.00) | -1.38 (0.2) | 20/476 | 4.73E-08 | 1.26 |
| Reactome | HSA-1445148 | Translocation of SLC2A4 (GLUT4) to the plasma membrane | 100% (0.02) | -1.87 (0.01) | 8/71 | 1.14E-05 | 1.21 |
| Reactome | HSA-195258 | RHO GTPase Effectors | 100% (0.00) | -2.31 (0) | 15/292 | 7.33E-07 | 1.2 |
| Reactome | HSA-168256 | Immune System | 91% (0.00) | -1.73 (0.01) | 48/1979 | 1.02E-12 | 1.18 |
| Reactome | HSA-447115 | Interleukin-12 family signaling | 100% (0.02) | -1.57 (0.05) | 7/56 | 3.55E-05 | 1.12 |
| Reactome | HSA-8950505 | Gene and protein expression by JAK-STAT signaling after Interleukin-12 stimulation | 100% (0.03) | -1.69 (0.03) | 6/37 | 5.92E-05 | 1.1 |
| Reactome | HSA-5663202 | Diseases of signal transduction by growth factor receptors and second messengers | 100% (0.00) | -1.84 (0.01) | 17/430 | 2.16E-06 | 1.05 |
| Reactome | HSA-449147 | Signaling by Interleukins | 88% (0.00) | -1.53 (0.1) | 16/453 | 2.11E-05 | 0.89 |
| Reactome | HSA-5663205 | Infectious disease | 96% (0.00) | -1.93 (0) | 24/917 | 3.89E-06 | 0.86 |
| Reactome | HSA-9658195 | Leishmania infection | 88% (0.02) | -1.84 (0.01) | 9/165 | 2.80E-04 | 0.81 |
| Reactome | HSA-109581 | Apoptosis | 100% (0.00) | -1.42 (0.17) | 9/175 | 4.20E-04 | 0.77 |
| Reactome | HSA-1280215 | Cytokine Signaling in Immune system | 84% (0.01) | -1.42 (0.17) | 19/706 | 6.08E-05 | 0.75 |

| Reactome | HSA-3858494 | Beta-catenin independent WNT signaling | 100% (0.02) | -1.72 (0.02) | 8/143 | 7.50E-04 | 0.74 |
| --- | --- | --- | --- | --- | --- | --- | --- |
| Reactome | HSA-194315 | Signaling by Rho GTPases | 100% (0.00) | -2.28 (0) | 18/672 | 1.30E-04 | 0.71 |
| Reactome | HSA-2029480 | Fcgamma receptor (FCGR) dependent phagocytosis | 100% (0.03) | -1.67 (0.03) | 6/89 | 3.10E-03 | 0.63 |
| Reactome | HSA-390466 | Chaperonin-mediated protein folding | 100% (0.03) | -1.79 (0.01) | 6/91 | 3.40E-03 | 0.62 |
| Reactome | HSA-9692914 | SARS-CoV-1-host interactions | 100% (0.03) | -1.64 (0.04) | 6/95 | 4.10E-03 | 0.6 |

Definition of abbreviations: GO=Gene Ontology; KEGG=Kyoto Encyclopedia of Genes and Genomes

Supplemental Table S4. Summary of Single Nucleotide Polymorphisms (SNPs) Associated with Protein Mediators of Endotoxin-Related Lung Function Change

| Protein Mediator | Single Nucleotide Polymorphism | Gene | Chromosome | Divide-Aggregate Composite-null Test p-value | Minor Allele | Alternative Allele | Minor Allele Frequency | Beta of Protein on SNP (p-value) | Beta of Annual Change in FEV-1 on SNP (p-value) |
| --- | --- | --- | --- | --- | --- | --- | --- | --- | --- |
| IGL c3728 | rs707944 | HLA-DQA1-AS1 | 6 | 3.4E-06 | A | T | 0.068 | -0.68 (4.07E-07) | -0.96 (4.53E-01) |
| IGL c3728 | rs9462216 | CPNE5 | 6 | 3.38E-06 | C | G | 0.245 | -0.38 (3.16E-06) | -0.03 (9.69E-01) |
| IGL c3728 | rs538292860 | CPNE5 | 6 | 3.81E-06 | A | ATG | 0.252 | -0.38 (3.31E-06) | -0.56 (4.48E-01) |
| IGL c3728 | rs236443 | CPNE5 | 6 | 4.53E-06 | C | G | 0.233 | -0.39 (2.86E-06) | -0.07 (9.23E-01) |
| IGL c3728 | rs236419 | CPNE5 | 6 | 3.86E-06 | T | C | 0.375 | -0.35 (2.59E-06) | -0.70 (3.03E-01) |
| IGL c3728 | rs186890 | CPNE5 | 6 | 4.29E-06 | C | A | 0.375 | -0.35 (2.81E-06) | -0.66 (3.34E-01) |
| IGL c3728 | rs34085629 | CPNE5 | 6 | 4.29E-06 | A | AG | 0.375 | -0.35 (2.81E-06) | -0.66 (3.34E-01) |
| IGL c3728 | rs9470391 | CPNE5 | 6 | 0.000004 | G | A | 0.227 | -0.40 (2.77E-06) | -0.28 (7.24E-01) |
| IGL c3728 | rs144332307 | CPNE5 | 6 | 2.78E-07 | C | G | 0.402 | -0.39 (1.14E-07) | -0.44 (5.16E-01) |
| IGL c3728 | rs6920224 | CPNE5 | 6 | 4.26E-07 | A | C | 0.335 | -0.40 (2.90E-07) | 1.24 (8.55E-02) |
| IGL c3728 | rs74646723 | CSMD1 | 8 | 4.82E-06 | C | T | 0.263 | -0.37 (3.68E-06) | -0.89 (2.20E-01) |
| IGL c3728 | rs78124869 | CSMD1 | 8 | 4.85E-06 | T | G | 0.278 | -0.37 (2.86E-06) | -1.04 (1.51E-01) |
| IGL c3728 | rs533142114 | LOC101927025 | 15 | 2.88E-07 | CA | C | 0.145 | -0.53 (3.45E-07) | -1.56 (9.97E-02) |
| IGL c3728 | rs62079898 | / | 18 | 4.92E-06 | C | G | 0.127 | -0.50 (1.37E-05) | -0.68 (5.05E-01) |
| HEL180 | rs11320829 | FARP2; STK25 | 2 | 3.21E-06 | GT | G | 0.373 | -0.35 (2.07E-06) | -1.23 (6.00E-02) |
| HEL180 | rs1918111 | / | 3 | 5.92E-07 | G | A | 0.093 | -0.56 (1.29E-06) | -0.57 (5.89E-01) |
| HEL180 | rs73198377 | / | 4 | 4.38E-06 | C | A | 0.227 | -0.38 (1.06E-05) | -2.01 (9.41E-03) |
| HEL180 | rs60941618 | / | 4 | 4.38E-06 | G | A | 0.227 | -0.38 (1.06E-05) | -2.01 (9.41E-03) |
| HEL180 | rs17218905 | / | 4 | 4.38E-06 | G | A | 0.228 | -0.38 (1.06E-05) | -2.01 (9.41E-03) |
| HEL180 | rs1903299 | / | 4 | 4.38E-06 | G | A | 0.228 | -0.38 (1.06E-05) | -2.01 (9.41E-03) |
| HEL180 | rs7928556 | NAV2 | 11 | 1.88E-06 | T | G | 0.399 | -0.35 (1.88E-06) | -1.23 (6.16E-02) |
| HEL180 | rs79378853 | NAV2 | 11 | 1.04E-06 | G | A | 0.384 | -0.36 (1.07E-06) | -1.19 (7.23E-02) |
| HEL180 | rs894558 | NAV2 | 11 | 3.67E-06 | C | T | 0.383 | -0.33 (4.47E-06) | -0.94 (1.42E-01) |
| HEL180 | rs4625554 | / | 12 | 2.96E-06 | A | G | 0.308 | -0.38 (2.59E-06) | -0.75 (2.97E-01) |
| HEL180 | rs4791475 | DNAH9A | 17 | 3.92E-06 | C | A | 0.383 | -0.37 (6.10E-06) | -0.02 (9.78E-01) |
| HEL180 | rs4792177 | DNAH9A | 17 | 2.26E-06 | A | C | 0.414 | -0.36 (3.00E-06) | -0.06 (9.32E-01) |
| HEL180 | rs34487424 | DNAH9A | 17 | 2.68E-06 | G | A | 0.488 | -0.33 (3.09E-06) | 0.00 (9.95E-01) |
| HEL180 | rs73241549 | / | 21 | 2.85E-06 | G | A | 0.137 | -0.48 (5.82E-06) | -0.81 (3.91E-01) |

Definition of abbreviations: FEV-1= forced expiratory volume in 1 second; HEL180= Epididymis luminal protein 180; IGL c3728= IGL c3728_light_IGKV4-1_IGKJ1


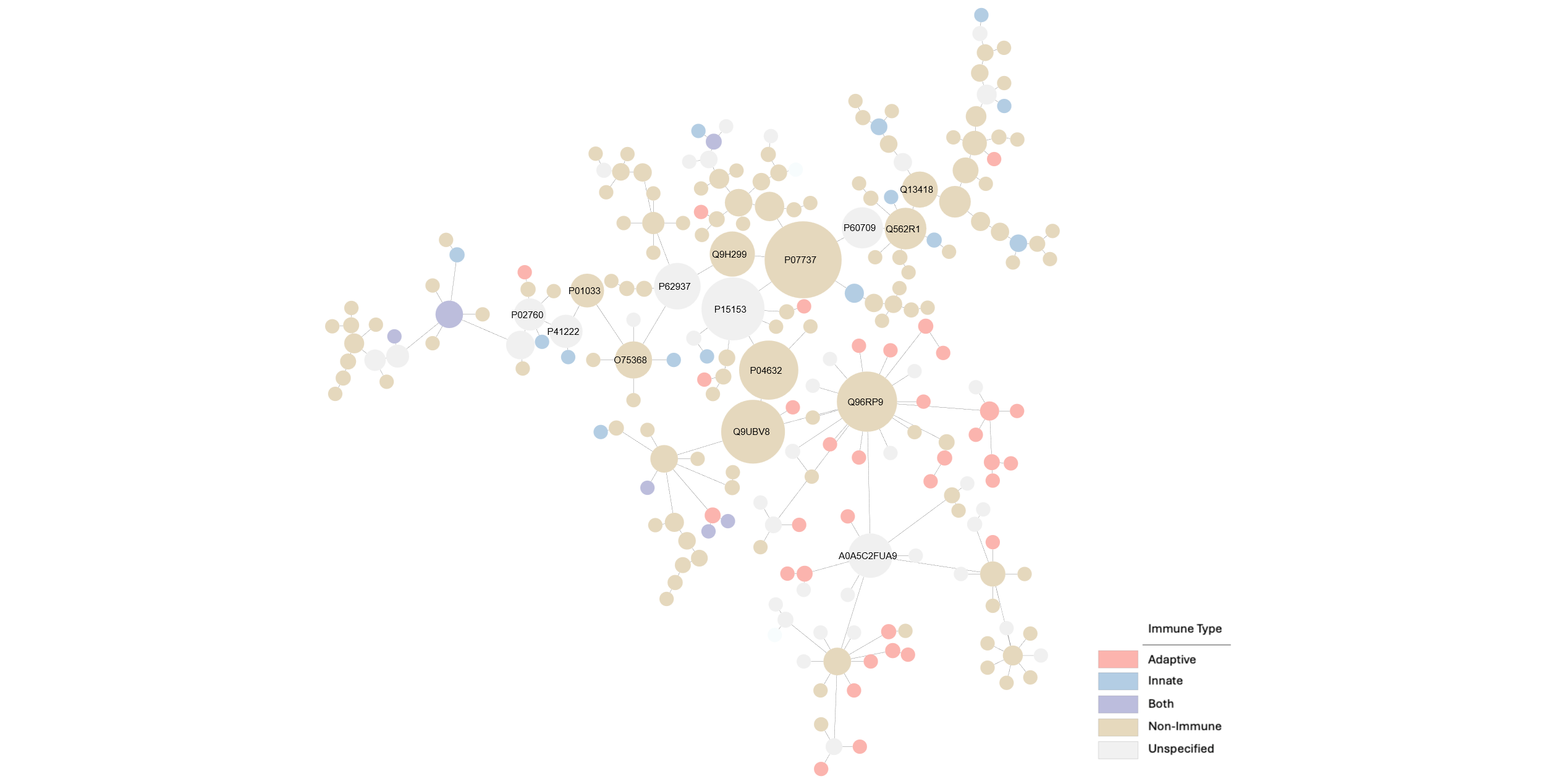


Supplemental Figure 1.

Protein–protein correlation network of differentially expressed proteins associated with occupation endotoxin exposure. The network was constructed using Prim’s maximum spanning tree. Only the top 15 proteins by betweenness centrality are labeled.


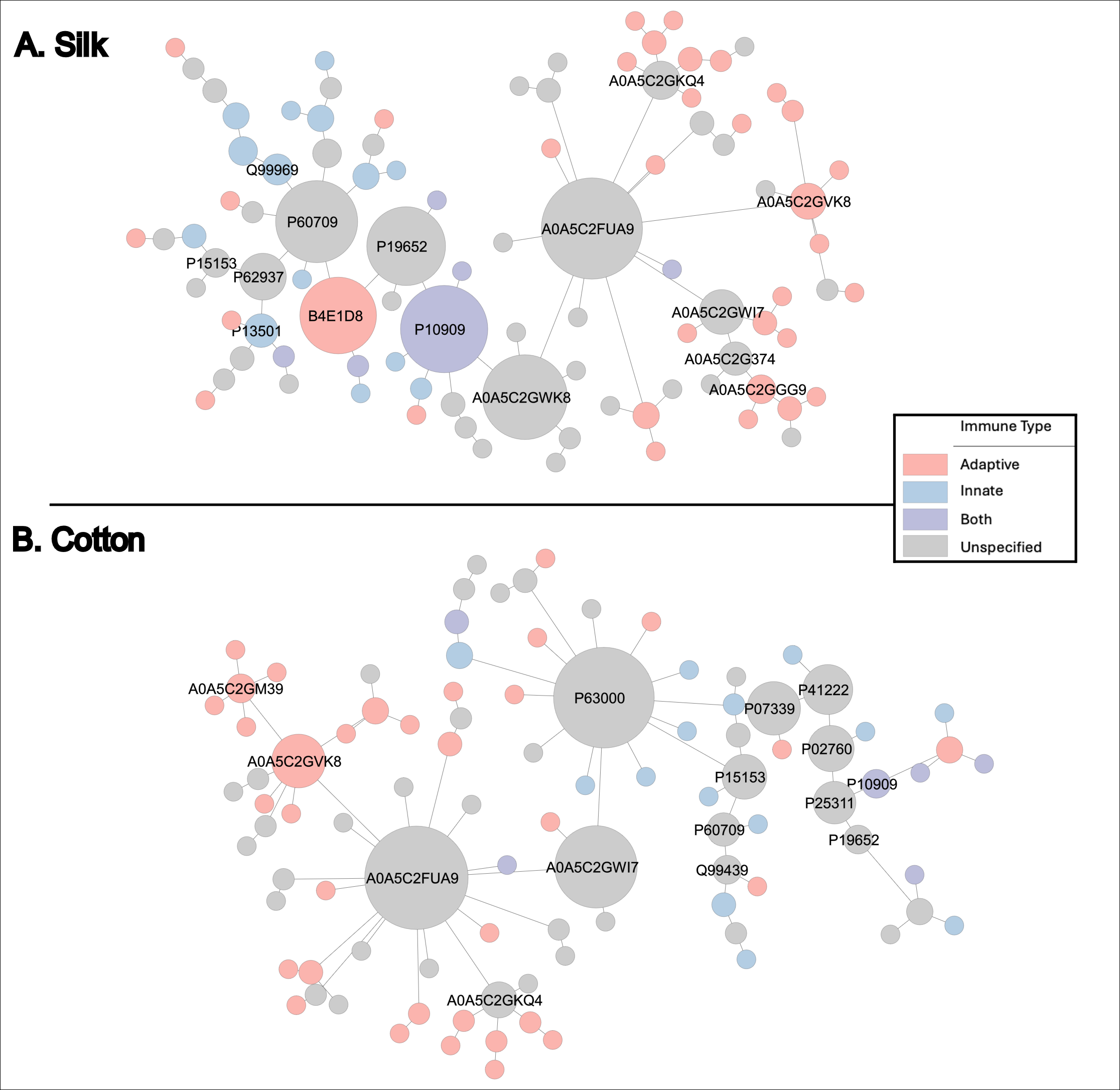


Supplemental Figure 2. Protein–protein correlation network of all immune-related differentially expressed proteins associated with occupation endotoxin exposure, stratified by occupation. The network was constructed using Prim’s maximum spanning tree. Only the top 15 proteins by betweenness centrality are labeled.

3. Supplementary Reference

1. Christiani DC, Eisen EA, Wegman DH, et al. Respiratory disease in cotton textile workers in the People's Republic of China. II. Pulmonary function results. Scand J Work Environ Health. 1986;12(1):46-50. doi:10.5271/sjweh.2175

2. Christiani DC, Ye TT, Wegman DH, Eisen EA, Dai HL, Lu PL. Cotton dust exposure, across-shift drop in FEV1, and five-year change in lung function. Am J Respir Crit Care Med. 1994;150(5 Pt 1):1250-1255. doi:10.1164/ajrccm.150.5.7952548

3. Christiani DC, Wang XR, Pan LD, et al. Longitudinal changes in pulmonary function and respiratory symptoms in cotton textile workers. A 15-yr follow-up study. Am J Respir Crit Care Med. 2001;163(4):847-853. doi:10.1164/ajrccm.163.4.2006063

4. Wang XR, Eisen EA, Zhang HX, et al. Respiratory symptoms and cotton dust exposure; results of a 15 year follow up observation. Occup Environ Med. 2003;60(12):935-941. doi:10.1136/oem.60.12.935

5. Wang XR, Zhang HX, Sun BX, et al. A 20-year follow-up study on chronic respiratory effects of exposure to cotton dust. Eur Respir J. 2005;26(5):881-886. doi:10.1183/09031936.05.00125604

6. Lai PS, Hang JQ, Valeri L, et al. Endotoxin and gender modify lung function recovery after occupational organic dust exposure: a 30-year study. Occup Environ Med. 2015;72(8):546-552. doi:10.1136/oemed-2014-102579

7. Comstock GW, Tockman MS, Helsing KJ, Hennesy KM. Standardized respiratory questionnaires: comparison of the old with the new. Am Rev Respir Dis. 1979;119(1):45-53. doi:10.1164/arrd.1979.119.1.45

8. Mehta AJ, Wang XR, Eisen EA, et al. Work area measurements as predictors of personal exposure to endotoxin and cotton dust in the cotton textile industry. Ann Occup Hyg. 2008;52(1):45-54. doi:10.1093/annhyg/mem061

9. Graham BL, Steenbruggen I, Miller MR, et al. Standardization of spirometry: 2019 update. An official American Thoracic Society and European Respiratory Society technical statement. Am J Respir Crit Care Med. 2019;200(8):e70–e88.

10. Zhao M, Wei L, Zhang L, et al. Proteomic biomarkers of long-term lung function decline in textile workers: a 35-year longitudinal study. J Expo Sci Environ Epidemiol. 2025;35(4):602-610. doi:10.1038/s41370-024-00721-7

11. Azur MJ, Stuart EA, Frangakis C, Leaf PJ. Multiple imputation by chained equations: what is it and how does it work?. Int J Methods Psychiatr Res. 2011;20(1):40-49. doi:10.1002/mpr.329

12. Thomas PD, Ebert D, Muruganujan A, Mushayahama T, Albou LP, Mi H. PANTHER: Making genome-scale phylogenetics accessible to all. Protein Sci. 2022;31(1):8-22. doi:10.1002/pro.4218

13. Rubinacci S, Hofmeister RJ, Sousa da Mota B, Delaneau O. Imputation of low-coverage sequencing data from

14. Prim RC. Shortest connection networks and some generalizations. Bell Syst Tech J. 1957;36(6):1389–1401.

15. Shannon P, Markiel A, Ozier O, et al. Cytoscape: a software environment for integrated models of biomolecular interaction networks. Genome Res. 2003;13(11):2498-2504. doi:10.1101/gr.1239303

150,119 UK Biobank genomes. Nat Genet. 2023;55(7):1088-1090. doi:10.1038/s41588-023-01438-3

16. Kanehisa M, Goto S. KEGG: Kyoto Encyclopedia of Genes and Genomes. Nucleic Acids Res. 2000;28(1):27–30. doi:10.1093/nar/28.1.27

17. Milacic M, Beavers D, Conley P, et al. The Reactome Pathway Knowledgebase 2024. Nucleic Acids Res. 2024;52(D1):D672-D678. doi:10.1093/nar/gkad1025

18. Massara GP, Di Matteo T, Aste T. Network filtering for big data: Triangulated maximally filtered graph. *J Complex Netw.* 2016;5(2):161–78.

19. Szklarczyk D, Kirsch R, Koutrouli M, et al. The STRING database in 2023: protein-protein association networks and functional enrichment analyses for any sequenced genome of interest. Nucleic Acids Res. 2023;51(D1):D638-D646. doi:10.1093/nar/gkac1000

20. VanderWeele TJ. Mediation Analysis: A Practitioner's Guide. Annu Rev Public Health. 2016;37:17-32. doi:10.1146/annurev-publhealth-032315-021402

21. Dai JY, Stanford JL, LeBlanc M. A multiple-testing procedure for high-dimensional mediation hypotheses. J Am Stat Assoc. 2022;117(537):198-213. doi:10.1080/01621459.2020.1765785

22. Liu Z, Shen J, Barfield R, Schwartz J, Baccarelli AA, Lin X. Large-Scale Hypothesis Testing for Causal Mediation Effects with Applications in Genome-wide Epigenetic Studies. J Am Stat Assoc. 2022;117(537):67-81. doi:10.1080/01621459.2021.1914634

23. Yang H, Liu Z, Wang R, Lai E-Y, Schwartz J, Baccarelli AA, Huang YT, Lin X. Causal mediation analysis for integrating exposure, genomic, and phenotype data. Annu Rev Stat Appl. 2024;12:337–360. doi:10.1146/annurev.statistics.040622.031653

24. Zhao Q, Wang J, Hemani G, Bowden J, Small DS. Statistical inference in two-sample summary data Mendelian randomization using robust adjusted profile score. Ann Stat. 2020;48(3):1742–1769.

25. Verbanck M, Chen CY, Neale B, Do R. Detection of widespread horizontal pleiotropy in causal relationships inferred from Mendelian randomization between complex traits and diseases. Nat Genet. 2018;50(5):693-698. doi:10.1038/s41588-018-0099-7

26. Rees JMB, Wood AM, Dudbridge F, Burgess S. Robust methods in Mendelian randomization via penalization of heterogeneous causal estimates. PLoS One. 2019;14(9):e0222362. Published 2019 Sep 23. doi:10.1371/journal.pone.0222362

27. Burgess S, Foley CN, Allara E, Staley JR, Howson JMM. A robust and efficient method for Mendelian randomization with hundreds of genetic variants. Nat Commun. 2020;11(1):376. Published 2020 Jan 17. doi:10.1038/s41467-019-14156-4

28. Burgess S, Thompson SG. Interpreting findings from Mendelian randomization using the MR-Egger method. Eur J Epidemiol. 2017;32(5):377-389. doi:10.1007/s10654-017-0255-x

29. Burgess, S., Butterworth, A., & Thompson, S. G. (2013). Mendelian randomization analysis with multiple genetic variants using summarized data. *Genetic epidemiology*, *37*(7), 658–665. https://doi.org/10.1002/gepi.21758
